# The thrombin receptor links brain derived neurotrophic factor to neuron cholesterol production, resiliency and repair after spinal cord injury

**DOI:** 10.1016/j.nbd.2021.105294

**Published:** 2021-02-05

**Authors:** Erin M. Triplet, Ha Neui Kim, Hyesook Yoon, Maja Radulovic, Laurel Kleppe, Whitney L. Simon, Chan-il Choi, Patrick J. Walsh, James R. Dutton, Isobel A. Scarisbrick

**Affiliations:** a Mayo Clinic Graduate School of Biomedical Sciences, Mayo Clinic Alix School of Medicine and the Mayo Clinic Medical Scientist Training Program Sciences Rochester, United States of America; b Department of Physical Medicine and Rehabilitation, Rehabilitation Medicine Research Center, United States of America; c Department of Physiology and Biomedical Engineering, Rochester, MN 55905, United States of America; d Department of Genetics and Cell Biology and Development, University of Minnesota, Minneapolis, MN 55455, United States of America

## Abstract

Despite concerted efforts to identify CNS regeneration strategies, an incomplete understanding of how the needed molecular machinery is regulated limits progress. Here we use models of lateral compression and FEJOTA clip contusion-compression spinal cord injury (SCI) to identify the thrombin receptor (Protease Activated Receptor 1 (PAR1)) as an integral facet of this machine with roles in regulating neurite growth through a growth factor- and cholesterol-dependent mechanism. Functional recovery and signs of neural repair, including expression of cholesterol biosynthesis machinery and markers of axonal and synaptic integrity, were all increased after SCI in PAR1 knockout female mice, while PTEN was decreased. Notably, PAR1 differentially regulated HMGCS1, a gene encoding a rate-limiting enzyme in cholesterol production, across the neuronal and astroglial compartments of the intact *versus* injured spinal cord. Pharmacologic inhibition of cortical neuron PAR1 using vorapaxar *in vitro* also decreased PTEN and promoted neurite outgrowth in a cholesterol dependent manner, including that driven by suboptimal brain derived neurotrophic factor (BDNF). Pharmacologic inhibition of PAR1 also augmented BDNF-driven HMGCS1 and cholesterol production by murine cortical neurons and by human SH-SY5Y and iPSC-derived neurons. The link between PAR1, cholesterol and BDNF was further highlighted by demonstrating that the deleterious effects of PAR1 over-activation are overcome by supplementing cultures with BDNF, cholesterol or by blocking an inhibitor of adenylate cyclase, Gαi. These findings document PAR1-linked neurotrophic coupling mechanisms that regulate neuronal cholesterol metabolism as an important component of the machinery regulating CNS repair and point to new strategies to enhance neural resiliency after injury.

## 1. Introduction

The limited capacity for repair following injury to the central nervous system (CNS) is one of the major challenges facing the field of neurology today. This deficit can be attributed to at least three factors: presence of growth-inhibitory factors, absence of pro-survival cues, and low intrinsic repair ability of CNS neurons (Anderson et al., 2018; David and Aguayo, 1981; Poplawski et al., 2020). In physiological conditions, the tight restriction of neurons to a mature, electrically active phenotype is highly beneficial, but becomes a detriment following insult with limited restoration of neural connections underlying poor functional recovery across neurological conditions. Understanding the molecular factors which lock CNS neurons into a mature but regeneratively silent state is critical to the development of therapies to foster neural repair in a temporally controlled manner.

The G-protein coupled receptor Protease Activated Receptor 1 (PAR1) is widely expressed in the CNS and is highly conserved across species from zebrafish to humans (Junge et al., 2004; Noorbakhsh et al., 2003; Striggow et al., 2001). In terms of neural development, PAR1 gene knockout mice show an accelerated pattern of myelination (Yoon et al., 2020; Yoon et al., 2015). With regard to neural injury, increases in PAR1 and its ligands occur with neuropathology, including stroke (Junge et al., 2003), traumatic brain injury (Itsekson-Hayosh et al., 2016), Alzheimer diseaseAlzheimer’s disease (Citron et al., 2016) and spinal cord injury (SCI) (Festoff et al., 2004; Radulovic et al., 2013; Radulovic et al., 2016; Yoon et al., 2013). Moreover, blocking the function of PAR1 improves outcomes in several of these conditions, with reductions in both astrogliosis and microgliosis (Nicole et al., 2005; Nishino et al., 1993; Radulovic et al., 2016; Suo et al., 2002; Yoon et al., 2020). In the context of traumatic SCI, we demonstrated that mice with constitutive PAR1 gene knockout show improved neurobehavioral recovery (Radulovic et al., 2016) and these effects were independently replicated (Whetstone et al., 2017).

Although considerable evidence now suggests that blocking the function of PAR1 is positioned to improve outcomes after neural injury, including neural preservation (Hamill et al., 2009; Radulovic et al., 2016; Rajput et al., 2014), little is known regarding the molecular mechanisms involved and the potential to impact repair. In particular, while it is now appreciated that activation of PAR1 can drive inflammatory responses in both the CNS (Nicole et al., 2005; Radulovic et al., 2016) and peripheral immune compartments (Vergnolle et al., 2004), far less is known regarding its neuron-specific roles. Indeed, despite its abundance in the CNS, there is very limited knowledge regarding physiological roles. To begin to address this knowledge gap, we applied whole genome RNA sequencing to identify transcriptional differences in the intact spinal cord between mature wild type and PAR1 knockout mice and the potential scope of PAR1’s regulatory actions after SCI. Coupled with an investigation of the impact of pharmacologic inhibition of PAR1 on neurite outgrowth and sprouting in cell culture, our findings suggest that PAR1 exerts direct regulatory actions on cellular machinery essential for nerve growth. Specifically, findings show for the first time that switching off PAR1 increases gene transcription pathways in the adult spinal cord that are involved in the production of lipids, particularly cholesterol, an essential component for membrane formation. Of particular interest, cholesterol availability is also a critical step in synaptogenesis (Mauch et al., 2001; Pfrieger, 2003). In murine cortical neuron cultures, switching off PAR1 increased neurite outgrowth and nerve sprouting in response to suboptimal BDNF, in part by increasing cholesterol biosynthesis. Together, these studies identify unique regulatory roles for PAR1 as a suppressor of CNS lipid and cholesterol production—in part by limiting the actions of BDNF—and that this can profoundly affect the capacity for neurite growth and repair. These findings point to pharmacological targeting of PAR1, already possible with FDA-approved Zontivity (Vorapaxar), as a possible promising strategy to enhance growth factor responsivity, cholesterol production and the intrinsic regenerative capacity of CNS neurons.

## 2. Materials and methods

### 2.1. Mice

Mice with PAR1 gene knockout (PAR1−/−, B6.129S4-F2rtm1Ajc/J, #002862) were backcrossed to C57BL6/J (#000664) for more than 50 generations (Burda et al., 2013; Radulovic et al., 2016; Yoon et al., 2020). All mice were from The Jackson Laboratory (Bar Harbor, ME). Age- and sex-matched C57BL6/J (PAR1+/+) mice served as controls. Mice were housed in specific-pathogen free facility with 12 h light/dark cycle in reusable cages with corncob bedding and paper rolls for environmental enrichment both before and after procedures. Food and water were provided *ad libitum* for the duration of the experiment. All animal experiments were carried out in accordance with NIH Guidelines for animal care and safety and approved by the Mayo Clinic Institutional Animal Care and Use Committee. Experimenters performing procedures and downstream analyses were blinded to genotype.

### 2.2. Lateral compression spinal cord injury

A total of 19 mice were used for histological and behavioral analyses in the 0.25 mm lateral compression model. Ten- to twelve-week old PAR1+/+ or PAR1− /− female mice underwent experimentally-induced traumatic SCI (*n* = 9–10 per genotype, with *n* = 6–7 for SCI, *n* = 3 uninjured controls, mean 19 ± 0.4 (SEM) g body weight across genotypes) as outlined in Zhang, 2008 (Plemel et al., 2008; Zhang and Liu, 2015). This is a widely used model of compression type injury of moderate severity resulting in robust astrogliosis, microgliosis, neuronal loss, and eventual incomplete recovery. Briefly, mice were deeply anesthetized with intraperitoneal administration of Xyalzine (10 mg/kg, Akorn Inc., Lake Forest, IL) and Ketamine (100 mg/kg, Hospira Inc., Fort Dodge, IA). The dorsal aspect of the spinal column was exposed by midline incision of approximately 10 cm, and spinal cord exposed by laminectomy of T8-T10 vertebrae using fine rongeurs. Durmont forceps with a 0.25 mm spacer was applied to laterally compress the T9 cord and surrounding dura for 14 s. Mice were allowed to recover on a heating pad with daily intraperitoneal injections of Buprenex for every 12 h for 72 h post-procedure following the initial pre-operative dose (0.05 mg/kg, Reckitt Benckiser Healthcare, England) and prophylactic administration of Baytril (10 mg/kg, Bayer Health Care, Shawnee Mission, KS). Bladders were manually evacuated twice daily until study endpoints, and mice were examined at these intervals for signs of infection or distress. Subjects demonstrating signs of distress, infection, or which were moribund were euthanized and excluded from the study. Experimenters were blinded to mouse genotype both prior to surgery and when conducting all post-operative follow-up and assessment, including histopathological analysis. Individual animals were treated as an experimental unit.

### 2.3. FEJOTA clip spinal cord injury

A total of 31 additional mice were used for RNAsequencing and histological and behavioral analysis in the FEJOTA clip SCI model. Twelve week old female PAR1+/+ or PAR1− /− mice (*n* = 8 per genotype for RNAsequencing, with *n* = 4 for SCI, *n* = 4 uninjured controls, and *n* = 7–8 for histology and neurobehavioral testing) were subjected to experimentally-induced contusion-compression injury by extradural application of an aneurysm clip with 8 g closing force (FEJOTA™ mouse clip) to the L2-L3 spinal cord (Joshi and Fehlings, 2002; Radulovic et al., 2015; Radulovic et al., 2016). The FEJOTA clip induces a more severe injury compared to lateral compression due to the contusion phase, increased compressive force and 60 s duration of insult. Laminectomy procedures and post-operative care was as described for the 0.25 mm lateral compression model. In all cases mice were randomized according to genotype prior to surgery and experimenters blinded to genotype throughout surgery and all subsequent experimental procedures.

### 2.4. Neurobehavioral outcome measurements

Mice were trained in the open field (LC and FEJOTA clip), ladder walk and inclined plane test (only FEJOTA clip) prior to surgery and a baseline for each mouse collected (0 dpi). The open field was used to evaluate seven categories of locomotor recovery using the Basso Mouse Scale (BMS), the day after surgery, and weekly thereafter until 30 dpi generating a maximum score of 9 (Basso et al., 2006). The ladder walk was used to evaluate sensorimotor coordination. Mice were observed crossing a horizontal ladder fitted with an angled mirror to view/record footfalls prior to surgery and on day 8, 15, 22 and 31 after injury. 4 mm rungs of the ladder were spaced between 7.5 (“easy”) or 16 (“hard”) mm apart, resulting in a maximum score of 51 or 30 positive events (correctly placed steps), respectively. The left and right hind limbs were scored for positive stepping events (plantar grasping, toe, skip), or foot-faults (miss, drag, spasm) and averaged to determine the total number of positive events for each mouse. The inclined plane test evaluates hind limb strength needed to maintain horizontal positioning on an inclining plane, with larger angles associated with better recovery. The last angle each mouse maintained a stance for 5 s before turning was recorded (Joshi and Fehlings, 2002). Inclined plane testing occurred after open field-testing weekly post-surgery starting 7 dpi. All behavior testing was scored by two independent observers, both blinded to genotype, and final score reported as mean of both independent scores.

### 2.5. Primary neuron cell culture

All cells were maintained in incubators at 37C and 5% CO2. Primary cortical neuron cultures were prepared from embryonic day 15 (E15) mouse pups. Meninges were removed from E15 brains and cortices microdissected in DMEM-F12 (Gibco 12,634,028) with 100 U/mL penicillin/streptomycin (Gibco 15,070,063) before being finely minced and digested with 0.25% trypsin (Gibco 15,090,046) diluted in Neurobasal media (Gibco 21,103,049) for 15 m at 37C. Cortical neurons were washed and manually triturated to create a single cell suspension in Neurobasal medium, 1× B27 supplement (Gibco 17,504,044), 1× Glutamax (Life Technologies 35,050–061), 100 U/mL penicillin/streptomycin (Gibco 15,070,063), 1× N2 supplement (Gibco A1370701 at 1×), and 10% (by volume) heat-inactivated fetal bovine serum (Gibco 10,082,147), and counted for plating in wells coated with poly-D-lysine (Sigma P6407–5MG, 0.1 mg/mL) and laminin (Fisher 23,017,015, 0.1 mg/mL). For immunocytochemistry, neurons were plated on PDL- and laminin-coated cover glass in 24-well plates at 150,000 cells/well, or for RNA and protein at 500,000 cells/well in PDL- and laminin-coated 6-well plates. Neurons remained in plating media overnight (12*h*) before media was changed to serum-free media (Neurobasal medium (Gibco 21,103,049), 1× B27 supplement (Gibco 17,504,044), 1× Glutamax (Life Technologies 35,050–061), 100 U/mL penicillin/streptomycin (Gibco 15,070,063)) for the duration of experiments. Immunostaining of representative cultures demonstrated that >94% of nuclei were positive for NeuN. Rare GFAP+ astrocytes were also visible; however, Iba1 staining was not detected.

### 2.6. SH-SY5Y culture

Human SH-SY5Y neurons (ATCC CRL-2266) were grown in serum-free media to avoid PAR activation by serum proteases. Cells were first cultured in full media at the time of plating (Neurobasal A medium, 1× B27 supplement, 1× N2 supplement, 1× Glutamax, 1× D-glucose (Sigma-Aldrich G8769), 1× sodium pyruvate (Corning 25–000-CI), 1× beta-mercapthoethanol (Gibco 21,985–023), and 100 U/mL penicillin/streptomycin) on plates coated in poly-L-ornithine (Sigma-Aldrich, P3655). 12 h after plating, cells were changed to minimal media (Neurobasal A, 1× N2 supplement, 1× Glutamax, 100 U/mL penicillin/streptomycin), at which time treatments were added.

### 2.7. Human induced pluripotent stem cell culture and differentiation

hiPSC line UMN PCBC16iPS (lab designation 9–1) was previously derived from neonatal human dermal fibroblasts (ATCC PCS 201–010) as described in (Ye et al., 2013) using CytoTune™ 1.0 non-integrating Sendai based vectors (ThermoFisher Scientific, Waltham, MA, USA). hiPSC line UMN MGS1–1473–2B6 (lab designation 2B6) was derived from primary conjunctival cells using the CytoTune™ 2.0 Sendai Reprogramming Kit (A16517, ThermoFisher Scientific) as previously described (Geng et al., 2017). hiPSC culture was performed essentially as previously described (Parr et al., 2016). Undifferentiated hiPSCs were maintained with Essential 8™ Medium (ThermoFisher Scientific A1517001) on tissue culture dishes coated with recombinant human vitronectin (Peprotech AF-140–09) with daily media changes. Cells were passaged with hypertonic citrate buffer (4.4 g/L Sodium Citrate Dibasic Monohydrate, 24 g/L Potassium Chloride) when 60–80% confluence was achieved. hiPSCs were reseeded at a 1:6 or 1:8 split ratio for maintenance and at a higher split ratio of 1:10 or 1:12 for differentiation on plasticware coated with poly-L-ornithine (Sigma-Aldrich P4957) and recombinant human laminin-521 (ThermoFisher Scientific A29248). The differentiation was initiated within 24 h post-passage, by exchanging the Essential 8™ medium on Day 1 of differentiation for Essential 6™ Medium (ThermoFisher Scientific A1516401) supplemented with 500 nM LDN-193189 HCl (a BMP inhibitor, Selleckchem S7507) and 100 nM BGJ398 (an FGFR inhibitor, Selleckchem S2183) as described in Walsh et al. (Walsh, 2020). For the remainder of the differentiation, the medium was changed every 24 h. Day 2; Essential 6™ medium with 4 μM CHIR99021 (a Wnt activator/GSK3 inhibitor, Tocris 4423), 20 ng/mL FGF2 (Peprotech 100–18B), and 500 nM A8301 (an Alk5/Smad inhibitor, Tocris 2939). Day 3; Essential 6™ with 100 nM BGJ398, 100 nM wntC59 (a Wnt/PORCN inhibitor, Tocris 5148), and 250 nM SAG (Smoothened agonist, Calbiochem 566,660). Day 4; Essential 6™ with 20 ng/mL FGF2 and 100 nM Retinoic acid (Sigma-Aldrich R2625). On Days 5 and 6 the Essential 6™ medium was supplemented with; 100 nM RA, 10 μM DAPT (Tocris 2634), and 10 ng/mL neurotrophin-3 (NT3, Peprotech 450–03) to stimulate committing to neuronal differentiation.

### 2.8. Cryopreservation of hiPSC-derived ventral spinal neurons

Day 7 hiPSC-derived ventral spinal neurons were dissociated with Accutase^™^ (ThermoFisher Scientific A11105–01) and resuspended into cryopreservation media consisting of Essential 6^™^ Medium supplemented with 10 ng/mL NT3, 1% human serum albumin (Sigma-Aldrich A1653), and 10% *v*/v dimethyl sulfoxide (DMSO, MilliporeSigma D8418).

### 2.9. Neurite outgrowth

Twenty-four h after plating primary neurons, media was replaced with fresh media and treatments applied, including 100 nM vorapaxar sulfate (Toronto Research Chemicals Inc., Toronto, CA), 1 or 10 ng/mL BDNF (Peprotech 450–02–2), 10 nM atorvastatin (Selleck Chemical, S2077), 100 μM PAR1 activating peptide TFLLR-amide (PAR1-AP, Peptides International), 10 nM pertussis toxin (Millipore Sigma P2980), and/or 10 μg/mL cholesterol (Thermo-Fischer C8667) and cells were harvested 24 or 72 h later. Control wells were treated with an equal volume of vehicle (200-proof ethanol for cholesterol, saline for BDNF and PAR1-AP and DMSO for all other treatments).

### 2.10. Neurite replacement scratch assay

To model nerve repair after injury, cortical neurons were grown to confluence for 5 days *in vitro* (DIV) when a scratch was made with a p1000 pipette tip down the center of each well. Culture media was changed immediately following scratch and treatments added as indicated in each experiment. Neurons were fixed for staining after an additional 5DIV.

All *in vitro* experiments were performed in biological triplicate and all experiments were repeated at least twice using independently derived cultures.

### 2.11. Histochemistry & cytochemistry

Immunostaining to quantify cellular antigens of interest in mouse samples was performed on 4% paraformaldehyde-fixed and paraffin-embedded spinal cords, as previously detailed (Radulovic et al., 2016; Yoon et al., 2017). For these studies, 2 mm blocks of spinal cord encompassing the lesion epicenter, in addition to two 2 mm block segments above and below, were embedded in a single paraffin block. Briefly, 6 μm sections were deparaffinized, followed by antigen retrieval (sodium citrate,10 mM, pH 6.0) and blocked with serum, followed by incubation with primary antibodies overnight. For immunofluorescence, sections were subsequently incubated with appropriate fluorochrome-conjugated secondary antibodies (Jackson ImmunoResearch Laboratories). 4^′^,6^′^-diamindino-2-phenylindole (DAPI, Vector Laboratories) was used as a nuclear stain and samples cover slipped with Fluoromount-G (Thermo-Fisher, 00–4958–02). For diaminobenzidine (DAB) staining, sections were incubated with species appropriate biotinylated secondary antibodies (Jackson Immunoresearch) and developed using avidin-biotin immunochemistry (Vectastain Elite ABC kit, PK-6100, Vector Laboratories) with 3,3^′^-diaminobenzidine tetrahydrochloride hydrate (DAB; D5637, Sigma-Aldrich) as the substrate. Nuclei were counterstained with methyl green (H-3402, Vector Laboratories, Inc.) and samples cover-slipped with DPX Mountant (06522, Sigma-Aldrich). Cultured cells were fixed with 2% paraformaldehyde, washed with phosphate-buffered saline (PBS) and blocked with 20% normal porcine serum (Thermo-Fisher, 31,890) before overnight primary antibody treatment and subsequent steps as described below for tissue immunofluorescence.

Immunostained specimens were imaged with an Olympus BX51 microscope and XM10 camera equipped with CellSens software 1.9 (Olympus, Center Valley, PA), under constant illumination. For primary neurons, five random fields per cover slip were imaged at the center and poles of each slide, or three fields centered on the scratch zone (defined by absence of DAPI staining) for the wound healing assay. All analysis was performed in ImageJ. Neurite outgrowth was quantified as total TUJ1+ area, normalized by number of DAPI+ nuclei in each field. Homer1+ punctae were counted with ImageJ Analyze Particles tool, and density calculated by normalizing total count to TUJ1+ neurite area. For HMGCS1 expression, the mean fluorescent intensity was calculated for the total cell area, reported as relative fluorescent units (RFU). For tissues, one section at the lesion epicenter and two sections from spinal segments both above and below injury zone were imaged and averaged for SCI mice. For uninjured mice, 7 segments corresponding to the same anatomical levels imaged in the SCI subjects were imaged and averaged. Analysis was performed in ImageJ. HMGCS1+ neurons (NeuN) were also assessed in ImageJ using the Pearson correlation in the Coloc2 plugin and the Binary Feature Extractor in the BioVoxxel plugin (https://imagej.net/BioVoxxel_Toolbox#Binary_Feature_Extractor). For HMGCS1 co-localization with astrocytes (GFAP), this was instead expressed as the percent of total GFAP+ area that was also positive for HMGCS1, due to inability to count individual astrocytes, particularly in the white matter.

Filipin staining was imaged on an Olympus BX51 microscope and XM10 camera equipped with CellSens software 1.9 (Olympus, Center Valley, PA). Cholesterol detection was performed using the cell-based cholesterol assay kit (Abcam, ab133116) according to manufacturer instructions. Briefly, cells were fixed with fixative for 10 min before being washed 3× for 5 min with wash buffer. Samples were incubated with filipin detection solution for 1 h at RT, then washed, and immediately imaged to prevent photobleaching.

### 2.12. Antibodies

### 2.13. Western blotting

Primary cortical neurons, prepared as for immunostaining were plated on 6-well plates at a density of 1.8 × 10^6^ cells/well for protein analysis. Protein was isolated by lysis of cells in cold lysis buffer and pelleting of cell debris by centrifugation. Protein concentration was determined by BCA, and samples (35 μg) were resolved on 4% to 20% Criterion TGX gels (Bio-Rad Laboratories, Hercules, CA) and electroblotted onto nitrocellulose. Membranes were then probed for HMGCS1 using the same antibody used for immunostaining, and then re-probed with an antibody recognizing β-actin (NB600–501, Novus Biologicals, Littleton, CO) to control for loading. Signal for each protein of interest in samples was detected on the same film using species appropriate horseradish peroxidase-conjugated secondary antibodies (GE Healthcare, Buckinghamshier, UK) and chemiluminescence (Pierce, Rockford, IL). The ROD of bands in each case was quantified with Image Lab 2.0 software (Bio-Rad Laboratories). The ROD of each protein of interest was normalized to that of actin and the mean and SEM of these raw values normalized to percent of the control samples for histograms.

### 2.14. Amplex Red cholesterol detection assay

An Amplex Red cholesterol fluorometric detection assay (Thermo-Fischer, A12216) was used for quantification of total cholesterol levels in cell culture. Cells were cultured as for immunostaining, treated as indicated in experimental set-up for a total of 72 h. For collection of samples, cell media was removed, and cells rinsed with PBS. Lipids were extracted with a solution of chloroform:methanol (at a ratio of 1:2) for 30+ minutes at 4 °C. Extraction buffer was collected and centrifuged at 10,000 rpm for 10 min at 4 °C to pellet any remaining cell debris; supernatants were collected and stored at −80 °C until assay could be performed. Amplex Red assay was carried out according to manufacturer instructions, with samples diluted 1:1 (a total of 25 μL from each well used per reaction) with 1X reaction buffer. Assay reaction was carried out on a 96 well plate, which was read using a Tecan Spark fluorometric plate reader for quantification.

### 2.15. Human spinal cord samples

To address the potential relevance of our findings to humans with SCI, we determined the expression of PAR1 and HMGCS1 by spinal cord neurons in 6 μm sections from a limited number of formalin-fixed, paraffin-embedded spinal cord blocks obtained at autopsy. Immunoperoxidase staining for each antigen was completed using sections from a non-SCI case, and from a case of traumatic SCI at 22 d (77 years, T5 motor vehicle accident) or 1247 d post injury (65 years female, C5 fall) (Radulovic et al., 2013; Scarisbrick et al., 2006). Antibodies used were HMGCS1 (1:1000, Thermo Fisher Scientific Cat# PA5–29488, RRID: AB_2546964) with biotinylated donkey anti-rabbit secondary (Jackson ImmunoResearch Labs Cat# 711–065–152, RRID:AB_2340593) and PAR1 (1:100, Santa Cruz Biotechnology Cat# sc-13,503, RRID: AB_2101175) with biotinylated donkey anti-mouse secondary (Jackson ImmunoResearch Labs Cat# 715–066–151, RRID:AB_2340788). Use of these spinal cord specimens was approved by the Mayo Clinic Institutional Review Board.

### 2.16. RNA sequencing

Spinal cords for RNA sequencing analysis were collected from salineperfused PAR1+/+ or PAR1− /− female mice 30 d after contusion-compression injury (FEJOTA clip) or uninjured controls, when mice were 120 d. Injured spinal cords were subdivided into 3 regions, with a 3 mm section collected from the epicenter of the injury, 3 mm of adjacent tissue rostral (“above”) and caudal (“below”) collected and analyzed separately. In the uninjured controls, the full 9 mm section corresponding to the same anatomical levels as the SCI group was collected and processed whole. Tissues were flash-frozen and stored at −80°C. RNA was purified using RNA STAT-60 (Tel-Test, Friendswood, TX). RNA libraries were prepared using 200 ng of total RNA according to the manufacturer’s instructions for the TruSeq RNA Sample Prep Kit v2 (Illumina, San Diego, CA). The concentration and size distribution of the completed libraries was determined using an Agilent Bioanalyzer DNA 1000 chip (Santa Clara, CA) and Qubit fluorometry (Invitrogen, Carlsbad, CA). Libraries were sequenced at 100 million reads per sample following Illumina’s standard protocol using the Illumina cBot and HiSeq 3000/4000 PE Cluster Kit. The flow cells were sequenced as 100bppaired end reads on an Illumina HiSeq 4000 using HiSeq 3000/4000 sequencing kit and HCS v3.3.20 collection software. Base-calling was performed using Illumina’s RTA version 2.5.2. mRNA-seq data was processed by the Mayo Bioinformatics Core Facility to identify genes with differential expression among groups. MAP-RSeq (Kalari et al., 2014) workflow (v1.2.1.3) was used to process mRNA-seq data, including read alignment, quality control, gene expression quantification and finally summarizing the data across samples. Paired-end reads are aligned by TopHat (v2.0.12) against the mouse genome build (mm10) using the bowtie1aligner (Langmead et al., 2009), and the gene counts were generated using Subread package (Anders et al., 2015) (v1.4.4). The workflow al so provides detailed quality control metrics across genes using the RSeQC (Wang et al., 2012) (v2.3.2). Differential expression analysis was performed with edgeR (Robinson et al., 2010) package (v2.6.2) to identify genes with altered expression. A cutoff for false discovery rate-adjusted *p*-value was set at 0.05 to determine the genes with significant expression change between conditions. The differential genes were further submitted to Ingenuity Pathway Analysis (IPA®) for pathway enrichment analysis and causal network analysis. For causal network analysis, only directed and experimentally observed interactions are used.

### 2.17. Quantitative PCR

Quantitative real-time reverse transcription PCR was used to quantify expression of transcripts for neuronal markers, cholesterol synthesis factors, and lipid regulatory factors in primary cortical and human SH-SY5Y neuron cultures. RNA was isolated using RNA STAT-60 (Tel-Test) and stored at −80°C. Amplification of the housekeeping gene Rn18s in all RNA samples was used to control for loading. Real-time PCR amplification in each case was accomplished with an iCycler iQ5 system (BioRad, Hercules, CA) using primers obtained from Integrated DNA Technologies (Coralville, IA) or Applied Biosystems (Grand Island, NY).

**Table:** All genes analyzed (HMGCS1, PAR1, PTEN) were normalized to Rn18S expression.

Gene Target	Company	Assay ID
Rn18S (mouse)	IDT	Mm.PT.51.3175696.g
Rn18S (human)	IDT	Hs.PT.39a.22214856.g
PAR1 (mouse)	Applied Biosystems	Mm00438851_m1
PTEN (mouse)	Applied Biosystems	Mm00477208_m1

### 2.18. Model generation

Summary figure of hypothesized models and summary figures were created with BioRender.com.

### 2.19. Experimental design and statistical analysis

Statistical analysis was performed in SigmaPlot (version 13). All data were expressed as mean ± standard error of the mean. For pairwise comparisons between two groups, two-tailed unpaired Student’s *t*-test was applied. Comparisons between multiple groups were made using One-Way ANOVA or Two-Way Repeated Measures ANOVA (for spinal cord injury histology at multiple levels) and tested for normality of the sample distribution with a Shapiro-Wilk test and equal variance with Brown-Forsythe test. Data sets which passed both (*P* > 0.05) were further assessed with the Student-Newman-Keuls pairwise multiple comparison procedure, with statistical significance set at *p* < 0.05. Data sets which failed Shapiro-Wilk testing underwent testing using ANOVA on Ranks procedure with Student-Newman-Keuls method multiple pairwise comparisons, again with significance set at *p* < 0.05. Associations between variables was tested by Pearson’s correlation coefficient (R), with significance set at *p* < 0.05. Specific tests used are indicated in figure legends. Quantification of results in all experiments was performed by experimenter without knowledge of genotype.

## 3. Results

### 3.1. PAR1 gene knockout increases expression of cholesterol synthesis machinery in the intact spinal cord

**Fig. 1.**
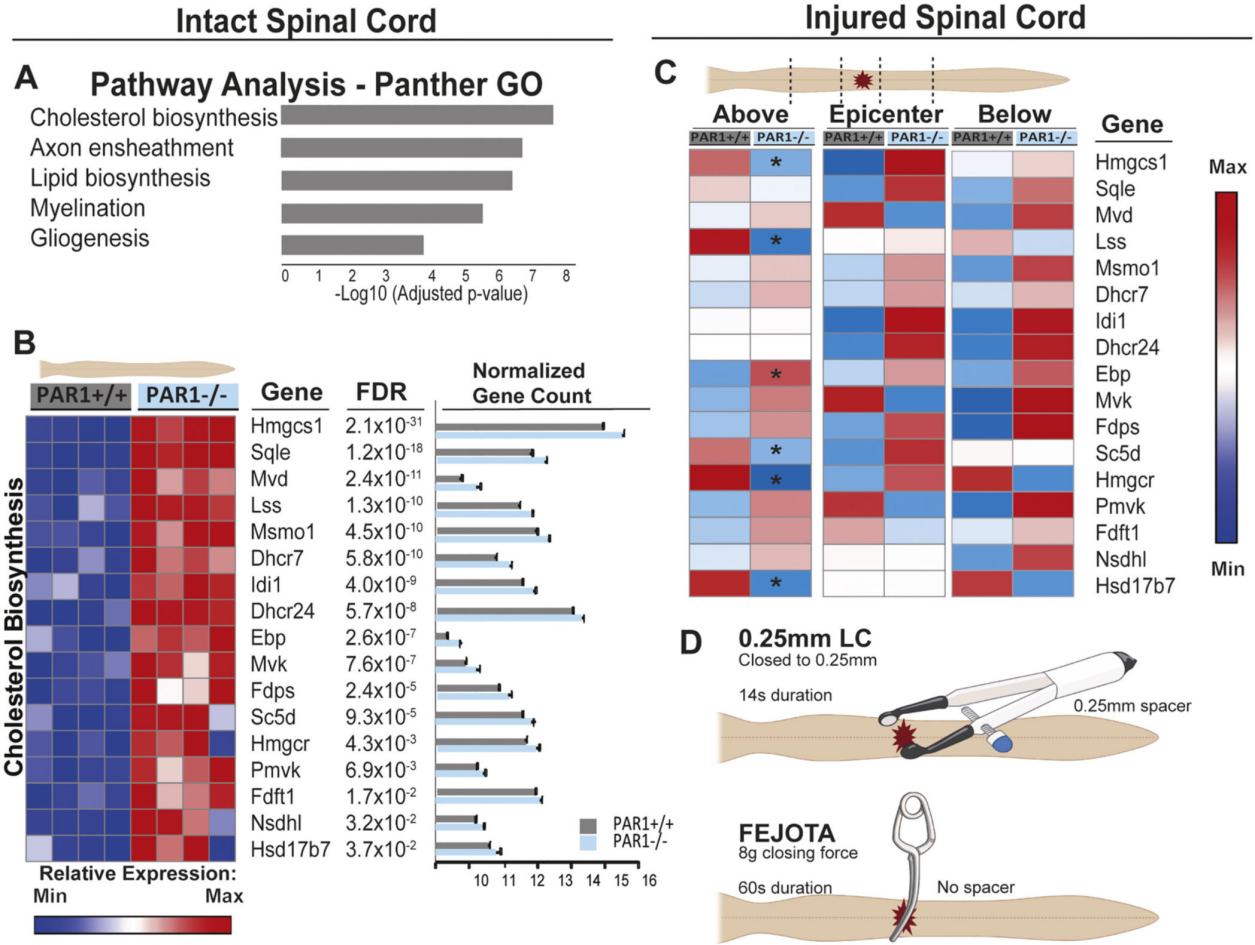
PAR1 knockout promotes increased expression of cholesterol synthesis intermediates in intact spinal cord. (A) GO pathway analysis of RNA sequencing of the spinal cord of 4 mice with constitutive PAR1 gene knockout and 4 age- and sex-matched wild type controls (120 d female mice) demonstrates that cholesterol biosynthesis was the most significantly differentially regulated GO term. (B) Heat map and histogram shows associated differential gene expression for key enzymes involved in the cholesterol synthesis pathway, all of which were increased with PAR1 gene knockout relative to wild type in intact spinal cord. Columns in heatmap represent individual animals. All cholesterol synthesis genes were significantly upregulated in the intact spinal cord, including HMGCS1 and HMGCR (the rate-limiting step in cholesterol biosynthesis) (FDR < 0.05, normalized gene counts and FDR values provided). (C) RNA sequencing of spinal segments spanning the injury epicenter, above or below 30 days after 8 g FEJOTA clip mediated SCI. Although dynamic changes were observed in genes encoding cholesterol synthesis enzymes across injury levels in PAR1−/− relative to PAR1+/+ (columns show average of 4 individual mice), only those marked with an asterisk reached FDR < 0.05 (data in Supplementary Fig. 1). (D) Schematic represents the two spinal cord injury models used throughout this paper, the 0.25 mm lateral compression (LC) in which a forceps with a 0.25 mm spacer is applied laterally to the cord for 14 s, and the FEJOTA contusion-compression model (FEJOTA), in which an aneurysm clip with an 8 g closing force is applied across the ventral-dorsal axis for 60 s.

To gain insights into mechanisms that may underpin the improvements in function observed after SCI in mice with constitutive PAR1 knockout compared to wild type comparators (Radulovic et al., 2016), we performed genome wide RNA sequencing of the intact and injured spinal cord (Fig. 1). First, Gene Ontology (GO) pathway analysis demonstrated that cholesterol biosynthesis was the most differentially expressed pathway between PAR1+/+ and PAR1−/− mice (Fig. 1A). Strikingly, examination of differentially expressed genes demonstrated that PAR1 knockout resulted in increased expression of every requisite enzyme in the *de novo* cholesterol biosynthesis pathway in the intact spinal cord compared to that of wild type mice (FDR < 0.05, Fig. 1B). For example, the spinal cord of PAR1 knockout mice showed increased expression of HMGCS1, a gene that encodes 3-hydroxy-3-methyglutaryl-CoA synthase 1, a rate limiting enzyme in cholesterol biosynthesis. This is of considerable interest, since the intact blood-brain barrier (BBB) is essentially impermeable to delivery of cholesterol from the periphery, such that the adult CNS becomes dependent on local cholesterol biosynthesis (Bjorkhem and Meaney, 2004; Zhang and Liu, 2015).

Given the important role of cholesterol to repair of neural membranes, we next sought to understand if the increased capacity for cholesterol production conferred by PAR1 gene knockout in the intact spinal cord was also observed after SCI. Parallel sequencing of RNA isolated at 30 dpi from the epicenter of 8 g FEJOTA clip contusion-compression injury, or spinal segments immediately above and below demonstrated dynamic changes in expression of several of the key intermediates in the cholesterol biosynthesis pathway in PAR1 knockouts compared to wild types. However, after controlling for false discovery rate, few of these changes reached the levels of statistical significance (Fig. 1C).

### 3.2. Expression of the cholesterol biosynthesis enzyme HMGCS1 by neurons and astrocytes of the intact and injured spinal cord

**Fig. 2.**
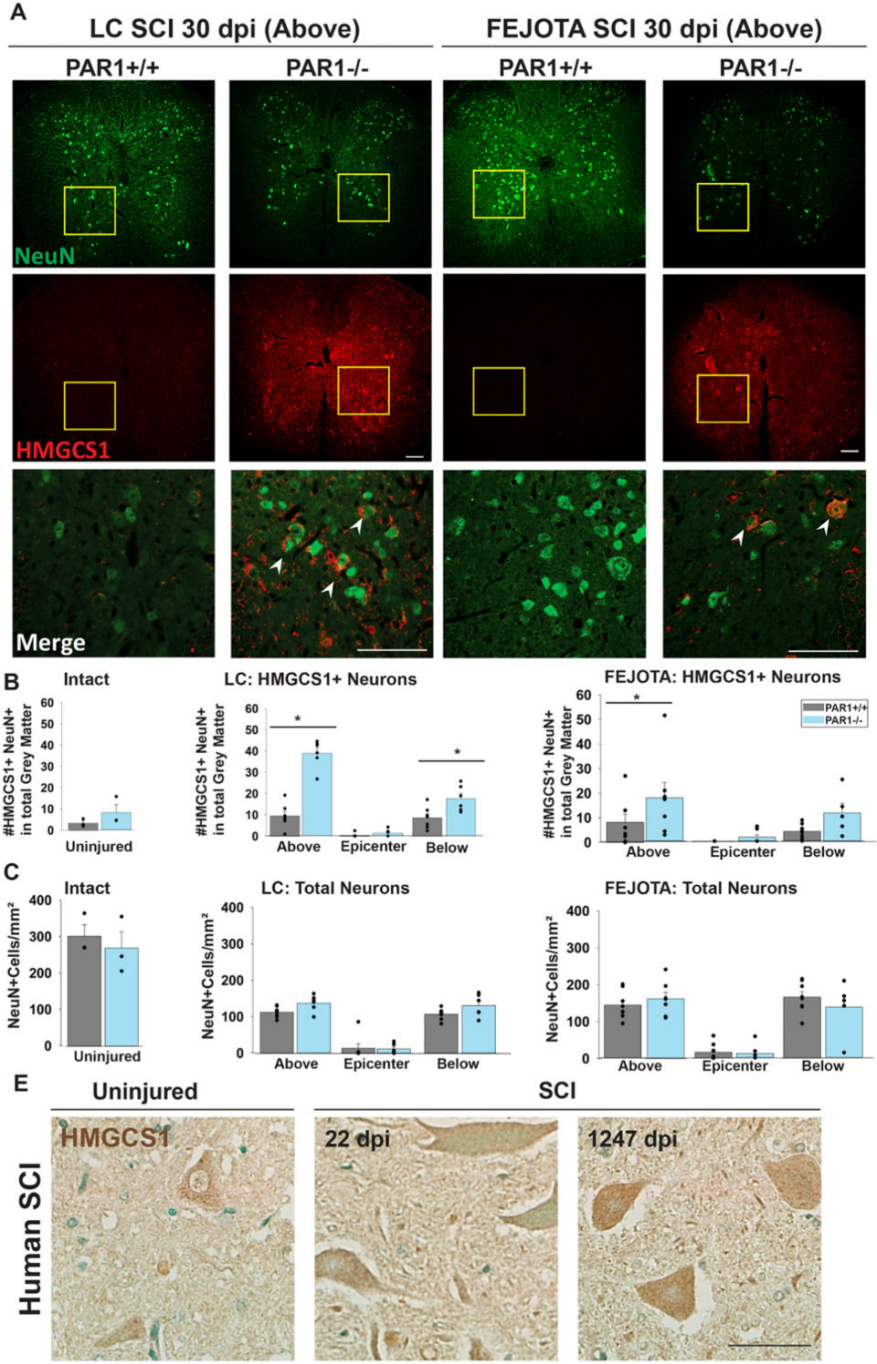
PAR1 knockout promotes increased expression of cholesterol synthesis intermediates by neurons in the injured spinal cord. (A) Immunofluorescent images show co-labeling for NeuN (neuron marker) and HMGCS1 in spinal segments above the injury epicenter 30 d after 0.25 mm lateral compression or FEJOTA clip SCI in wild type (PAR1+/+) and PAR1−/− mice. The merged images show higher power views of boxed areas to demonstrate increases in neuronal HMGCS1 expression in PAR1−/− compared to controls after SCI (arrow heads indicate HMGCS1 + NeuN+ ventral horn neurons). (B) Histogram shows significant elevations in the number of HMGCS1+ neurons 30 dpi in spinal segments above and below the injury epicenter (*p* < 0.001 Above, *p* = 0.002 Below) in LC and Above (*p* = 0.04) in FEJOTA. Few HMGCS1+ neurons are present in the intact spinal cord (uninjured). (C) In contrast, the total number of neurons in the spinal cord did not significantly differ by genotype in either model. (D) Expression of HMGCS1 by ventral horn motoneurons was also documented in the intact in human spinal cord and at subacute and chronic time points after traumatic SCI. Differences between genotypes were measured by two-tailed Student’s *t*-test (uninjured) or Two-Way Repeated Measures ANOVA with Student-Newman-Keuls pairwise comparisons (SCI), *p < 0.05. For immunostaining, *n* = 7 PAR1+/+ and 6 PAR1−/− female mice for LC, *n* = 8 PAR1+/+ and 7 PAR1−/− for FEJOTA, *n* = 3 each genotype for uninjured. Co-expression between uninjured and SCI (average of all segments) was also assessed by Two-Way ANOVA; differences between intact and SCI were not significant in either genotype. Scale bar = 100 μM A, 50 μM D.

**Fig. 3.**
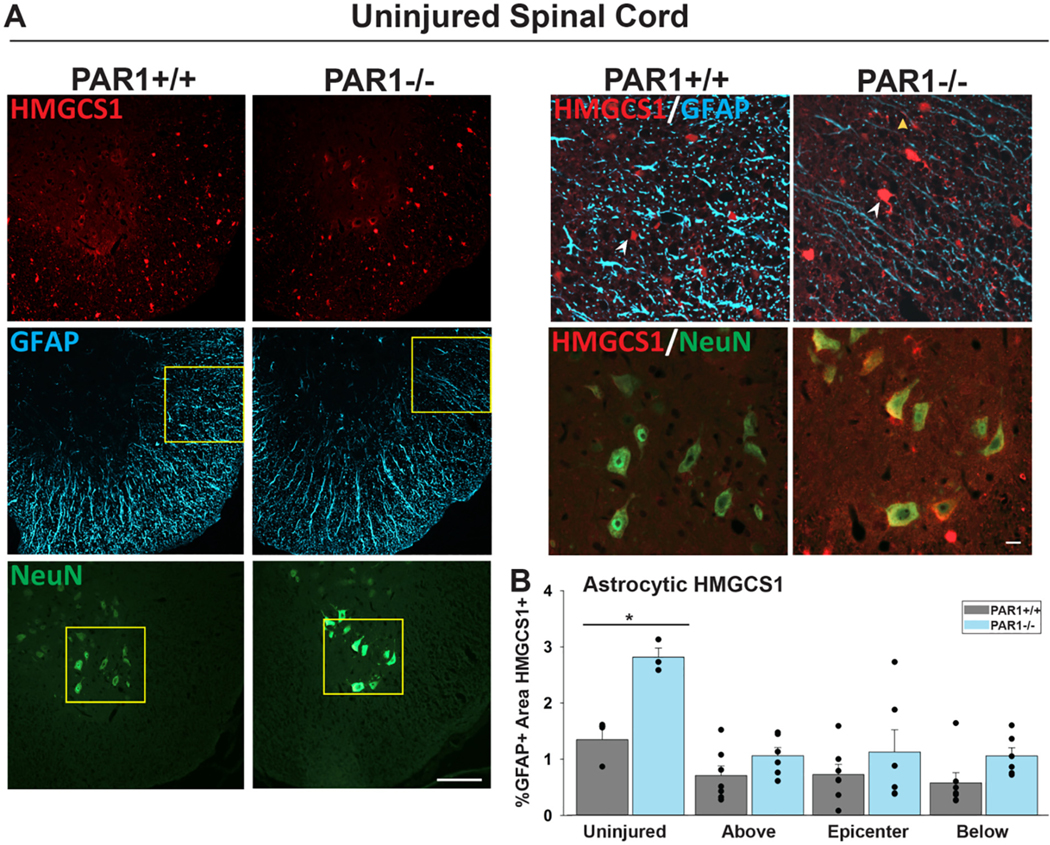
PAR1 knockout promotes increased expression of the cholesterol synthesis intermediate HMGCS1 by astrocytes in the intact spinal cord. (A) Immunofluorescent images show co-labeling for HMGCS1, GFAP (astrocyte marker), and NeuN (neuron marker) in the ventrolateral region of the intact spinal cord. (B) Histogram shows quantification of the percentage of total GFAP+ area also positive for HMGCS1. Astrocytes in PAR1−/− mice expressed 2-fold more HMGCS1 compared to wild-type mice (*p* = 0.007, two-tailed Student’s t) in the intact cord. Astrocyte HMGCS1 expression was decreased at 30 dpi across the injury epicenter and spinal segments above and below compared to the intact cord in both PAR1+/+ and PAR1−/− mice (LC SCI tissue averaged across all levels, *p* = 0.02 for PAR1+/+ and *p* < 0.001 for PAR1−/− by Two-Way ANOVA). There was a trend toward increased co-expression of HMGCS1 in PAR1−/− mice in neurons (*p* = 0.07) in the intact spinal cord, with increased neuronal expression of HMGCS1 following SCI (see Fig. 2). Arrow heads point to non-GFAP+ white matter glia (likely oligodendrocytes) also expressing HMGCS1, small yellow arrow denotes area of GFAP-HMGCS1 co-expression. Difference between genotypes assessed by two-tailed Student’s t-test (uninjured) and Two-Way Repeated Measures ANOVA (levels within SCI). Co-expression between uninjured and SCI (average of all segments) was also assessed by Two-Way ANOVA. *p < 0.05, *n* = 3 each for uninjured, *n* = 7 PAR1+/+ and *n* = 6 PAR1−/− LC SCI mice. Scale bar = 100 μM.

Since PAR1 knockout drove strong increases in cholesterol biosynthesis enzymes in the intact spinal cord and showed important trends toward increases in the injury epicenter and below 30 dpi (Fig. 1A–C), we next applied immunofluorescence co-labeling approaches to investigate any cell type specific changes in the 0.25 mm LC (LC) and FEJOTA clip contusion-compression (FEJOTA) models of SCI (Fig. 1D). In the intact CNS, neurons are thought to be largely dependent on astrocytes for delivery of cholesterol (Ferris et al., 2017; Goritz et al., 2002; Mauch et al., 2001) and therefore these neural types became the focus of our study. We investigated HMGCS1 as a key marker for cholesterol biosynthesis and co-labeled with either the neuronal marker NeuN (Fig. 2), or GFAP as a marker for reactive astrocytes (Fig. 3). Given the heterogeneity of pathological mechanisms and repair processes ongoing in spinal cord segments above, at, or below the injury epicenter, we examined each region as a potentially unique microenvironment. At 30 d following LC and FEJOTA SCI, we observed an increase in the number of HMGCS1+ spinal cord neurons compared to the intact (uninjured) cord in both PAR1+/+ and PAR1−/− mice. PAR1−/− mice demonstrated significantly greater numbers of HMGCS1+ neurons in the 4 mm of spinal cord above (*n* = 6–7, total *df* = 38, *p* < 0.001) and below (*n* = 6–7, total *df* = 38, *p* = 0.002, Two-Way Repeated Measures ANOVA) the injury zone compared to wild type mice in the LC model as well as above (*n* = 7–8, total *df* = 42, *p* = 0.04, Two-Way Repeated Measures ANOVA) in the FEJOTA model (Fig. 2A–C). The number of HMGCS1 + NeuN+ neurons was almost 4-fold higher in spinal segments above and 2-fold higher in spinal segments below the site of injury in PAR1−/− mice following LC SCI. There was no difference in overall numbers of total NeuN+ neurons across the dorsal and ventral areas of grey matter in PAR1+/+ compared to PAR1−/− mice at any level examined (Fig. 2C. LC: *n* = 6–7, total *df* = 38, *p* = 0.40 Above, *p* = 0.88 Epicenter, and *p* = 0.08 Below. FEJOTA: *n* = 7–8, total *df* = 42, *p* = 0.41 Above, *p* = 0.85 Epicenter, and *p* = 0.23 Below. Two-Way Repeated Measures ANOVA), nor was there any significant difference in spared tissue volume at any level as measured using automated Cavalieri estimator (LC: PAR1+/+ Epi spared volume was 0.60 ± 0.15 *vs* PAR1−/− 0.38 ± 0.12 mm^3^, FEJOTA: PAR1+/+ Epi spared volume was 0.89 ± 0.46, PAR1−/− 1.39 ± 0.58; *p* = 0.79 between genotypes in LC model, *p* = 0.55 in FEJOTA model, Two-Way Repeated Measures ANOVA) (Kim et al., 2021). Together, these data suggest that loss of PAR1 function in the context of SCI results in increases in expression of the cellular machinery required for cholesterol biosynthesis, including expression by neurons. Strengthening the potential significance of cholesterol production by neurons after SCI, we confirmed neuronal expression of HMGCS1 in the context of human cases of SCI. Neuronal HMGCS1 expression was observed both in the uninjured cord and at subacute (22 d) and a chronic time point (1247 d) after SCI (Fig. 2D).

In the intact spinal cord, HMGCS1 expression in GFAP+ astrocytes was 2-fold higher in PAR1−/− mice compared to PAR1+/+ (*n* = 3 each, *df* = 4, *t* = −5.054, *p* = 0.007, two-tailed Student’s *t*-test) (Fig. 3). At 30 dpi, GFAP+ astrocytes demonstrated decreased expression of HMGCS1 in the injured cord (all levels) compared to the intact cord in both genotypes (*n* = 3 uninjured, 6–7 LC SCI, total *df* = 18, *p* = 0.02 for PAR1+/+, *p* < 0.001 for PAR1−/− by Two-Way ANOVA). Astrocytic expression was not significantly different between genotypes at 30 dpi (*n* = 6–7, total *df* = 38, *p* = 0.11, Two-Way Repeated Measures ANOVA), but trended to increased expression in PAR1−/− at all levels (Fig. 3). Understanding PAR1 regulated HMGCS1 expression in the subpopulation of white matter glia not GFAP+, presumably of the oligodendrocyte lineage, was not pursued in the current study but will be an important line of follow up study.

**Fig.4.**
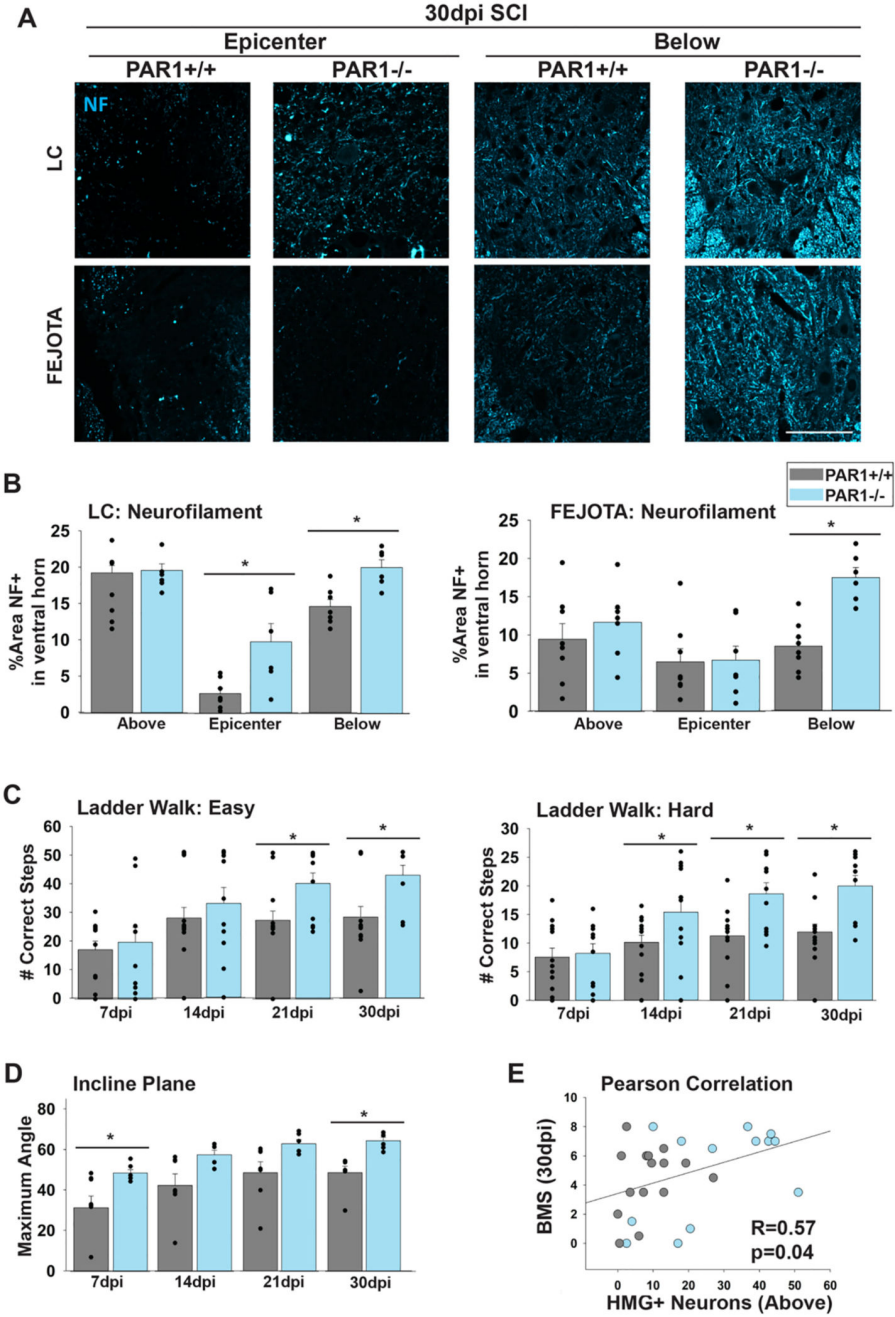
Neuronal cholesterol production in PAR1 knockouts is associated with signs of improved neural recovery following SCI. (A) Immunofluorescent images show labeling for Neurofilament (NF) in the ventral horn SCI Epicenter and Below in wild type (PAR1+/+) and PAR1−/− 30 d 0.25 mm LC and FEJOTA clip SCI. Images and associated histograms (B) show PAR1−/− spinal cords have increased staining for neurofilament heavy chain, a marker of axons, in both the injury Epicenter (*p* = 0.002) and Below (*p* = 0.01) for LC and Below (*p* = 0.002) for FEJOTA clip compared to PAR1+/+ mice. PAR1−/− mice show an accelerated pattern of motor recovery in ladder walk with steps spaced 7.5 (“easy”) or 16 (“hard”) mm apart, resulting in a maximum score of 51 or 30 correctly placed steps (C) and in inclined plane testing, measured by maximal angle at which subject was able to maintain position (D). (E) The number of HMGCS1+ neurons in the grey matter Above was also identified as a histological marker of functional recovery positively correlating with improved Basso Mouse Scale scores at 30 dpi (Pearson Correlation, R = 0.57, *p* = 0.04). Differences between genotypes were measured by Two-Way Repeated Measures ANOVA with Student-Newman-Keuls pairwise comparisons, *p < 0.05, *n* = 7 PAR1+/+ and 6 PAR1−/− female mice for LC, *n* = 8 PAR1+/+ and 7 PAR1−/− for FEJOTA clip. Scale bar = 100 μM.

PAR1 knockout mice show improved recovery of function after SCI in multiple models of injury examined to date, including the 8 g FEJOTA clip model of contusion-compression injury (Radulovic et al., 2016) and a thoracic contusion model (Whetstone et al., 2017). Reductions in inflammation and astrogliosis and signs of preserved neural integrity were documented in each case. Given the significant increases in HMGCS1 in neurons of the injured PAR1 knockout spinal cord (Fig. 2), we investigated any correlations with signs of neural recovery (Fig. 4). Here, using spinal cord samples from both LC and FEJTOA SCI models we identified a preservation of staining for neurofilament proteins in PAR1 knockouts relative to wild type mice at 30 dpi, in addition to improvements in BMS scores. Neurofilament is a marker of neuronal processes that is abundant in motor neuron axons (Tsang et al., 2000), and was greater in the ventral horn of PAR1−/− compared to wild type mice at 30 dpi. PAR1−/− mice demonstrated significantly more area positive for neurofilament in both the injury epicenter (LC: *n* = 6–7, total *df* = 38, *p* = 0.002, Two-Way Repeated Measures ANOVA) and in the 4 mm of spinal cord below (LC: *n* = 6–7, total *df* = 38, *p* = 0.01, FEJOTA: *n* = 7–8, total *df* = 42, *p* = 0.002, Two-Way Repeated Measures, Fig. 4A–B). Axon density above the compression site did not differ between genotypes. In the FEJOTA clip model, we also found that mice lacking PAR1 exhibited accelerated recovery of function in multiple neurobehavioral testing modalities, including ladder walk and inclined plane^1^; both tests of motor function (Fig. 4C–D) (Radulovic et al., 2016). Using the Pearson coefficient of correlation, we identified a significant relationship between neuronal HMGCS1 expression and motor outcomes (Fig. 4E). The number of neurons positive for HMGCS1 in the region above the injury site was positively correlated with functional status as measured by Basso Mouse Scale (BMS) score at 30 dpi (*n* = 28, *R* = 0.57, *p* = 0.04, Fig. 4E). Together, these findings suggest cholesterol synthesis enzymes are highly elevated in neurons after SCI in mice lacking PAR1 relative to wild type mice and that this is associated with improvements in functional status and neural elements, including neurofilament density.

### 3.3. PAR1 inhibition enhances BDNF-mediated cholesterol synthesis and neurite outgrowth in vitro

**Fig. 5.**
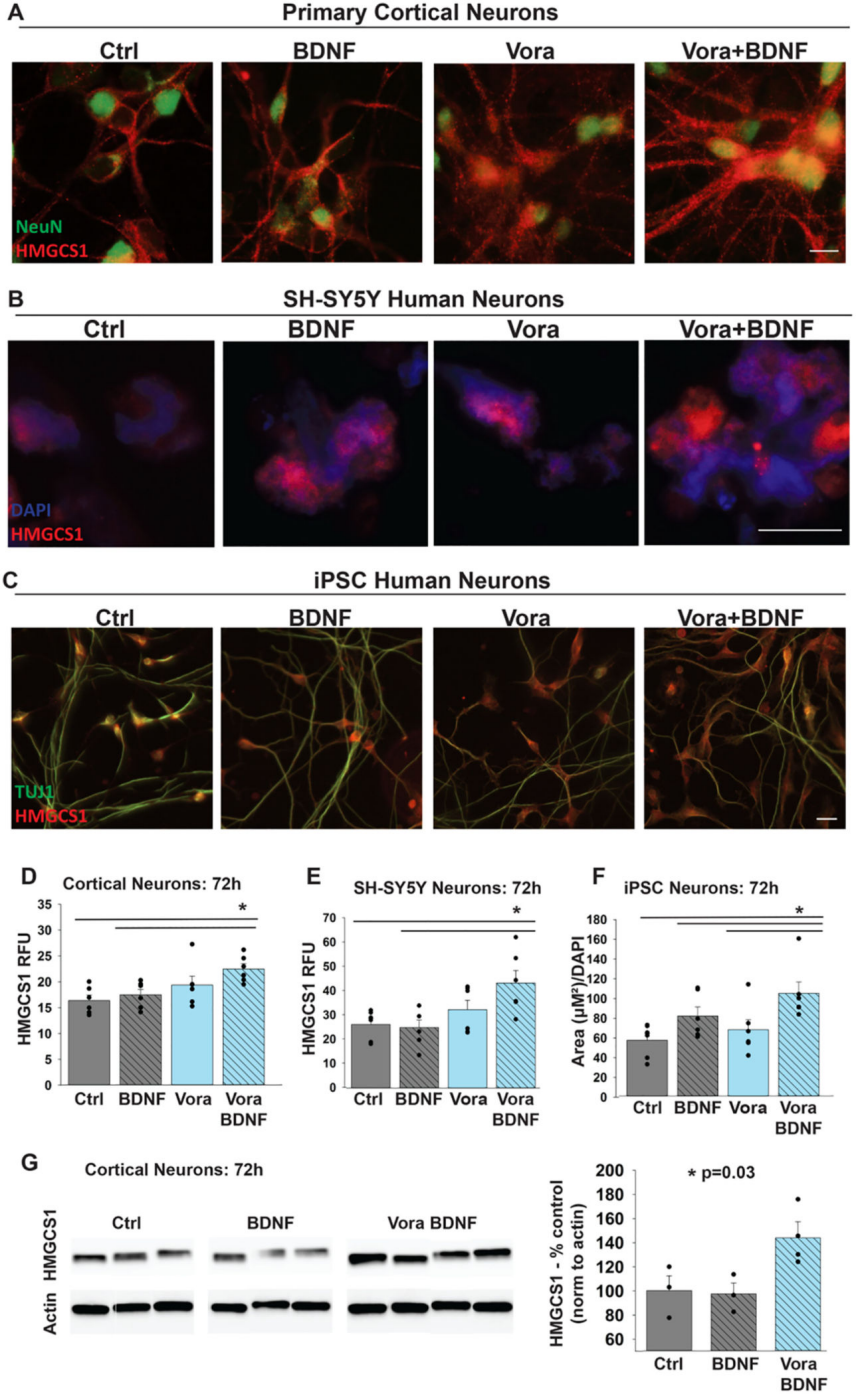
PAR1 inhibition augments BDNF-mediated HMGCS1 production by murine and human neurons *in vitro*. Immunofluorescent (IF) images show staining for HMGCS1 (red) and neuronal marker NeuN (green) in primary murine cortical neurons treated with low-dose BDNF (1 ng/mL), the PAR1 inhibitor vorapaxar (100 nM) or a combination for 72 h. (D) The intensity of HMGCS1 in relative fluorescence units (RFU) was increased by Vora+BDNF at 72 h (*p* = 0.01 compared to control, *p* = 0.03 relative to BDNF alone). HMGCS1 staining was also performed in human neurons, in both the SH-SY5Y cell line (B) and induced pluripotent stem cells differentiated into a spinal cord neuron phenotype (C) after treatment with vorapaxar (100 nM SH-SY5Y, 50 nM iPSC), BDNF (1 ng/mL), or combination for 72 h. Combination Vora+BDNF increased HMGCS1 fluorescence intensity relative to control and BDNF alone (D) (*p* == 0.01 for each) in SH-SY5Y neurons (E) and increased HMGCS1 expression relative to control, BDNF, and vorapaxar alone (*p* = 0.02, 0.01, and 0.01, respectively) in iPSC-derived human spinal cord neurons (F). (G) Increased HMGCS1 protein levels in cortical neurons with combined vorapaxar and BDNF over control or BDNF alone confirmed by Western blotting (*p* = 0.03 overall, paired post-tests not statistically significant). Statistical significance determined by One-Way ANOVA on Ranks (iPSC neurons, due to unequal variance) or One-Way ANOVA (all other analyses) with Student-Newman-Keuls pairwise comparisons, *p < 0.05, *n* = 6 per condition for immunostaining, cortical neuron data derived from at least 2 independent preps. Scale bar = 10 μM in A, 20 μM in B,C.

Based on the overall preponderance of HMGCS1 expression in neurons compared to astrocytes in both the intact and injured spinal cord (Figs. 2 and 3), we next focused efforts on determining the potential significance of PAR1 loss-of-function to neuronal cholesterol production in the context of neurite outgrowth in cell culture (Fig. 5). First we determined the impact of PAR1 inhibition on neuronal cholesterol production in primary murine cortical neurons *in vitro* by application of vorapaxar, an FDA approved PAR1 small molecule inhibitor, to the culture media. Given our recent findings that PAR1 inhibition increases sensitivity of OPCs to brain derived growth factor (BDNF) and the established role of BDNF in neuron development, neurite outgrowth, and neuronal cholesterol production (Cohen-Cory et al., 2010; Lucini et al., 2018; Numakawa et al., 2018; Spagnuolo et al., 2018; Suzuki et al., 2007) we next investigated any interaction between neuronal PAR1 inhibition and BDNF by treatment of primary cortical neurons with a low dose of BDNF (1 ng/mL) alone. The 1 ng/mL concentration of BDNF examined was considered a low dose since 10 ng/mL is established to promote optimal neurite growth (Santos et al., 2016). We posit a low dose of BDNF is therapeutically relevant mirroring reductions in the availability of growth factors at sites of neural injury (Fan et al., 2018; Widenfalk et al., 2001). A 72 h treatment with vorapaxar or BDNF alone did not result in increases in cortical neuron expression of HMGCS1. However, combined vorapaxar+BDNF resulted in a 1.4-fold increase in HMGCS1 expression by immunofluorescence (Fig. 5A,D, *n* = 6 each, total *df* = 23, *p* = 0.01, One-Way ANOVA) and by Western blotting (Fig. 5G, *n* = 3–4 each, total *df* = 9, overall *p* = 0.03, One-Way ANOVA).

The combination BDNF-vorapaxar also triggered increased neuronal expression of cholesterol synthesis machinery in human neurons. SH-SY5Y neurons treated for 72 h with combined vorapaxar and low-dose BDNF increased HMGCS1 immunofluorescence 1.6-fold (Fig. 5B,E, *n* = 6 each, total *df* = 23, *p* = 0.01, One-Way ANOVA). Similarly, human neurons derived from induced pluripotent stem cells (iPSC) with subsequent differentiation into a spinal cord neuron phenotype also demonstrate significant increases in HMGCS1 expression when treated with vorapaxar with BDNF—the combination resulted in a 2-fold increase in HMGCS1 (Fig. 5C,F, *n* = 6 each, *df* = 3, *p* = 0.02, One-Way ANOVA on Ranks). Together, these findings highlight the potential translatability of PAR1-inhibition as a method to enhance neurite growth.

**Fig. 6.**
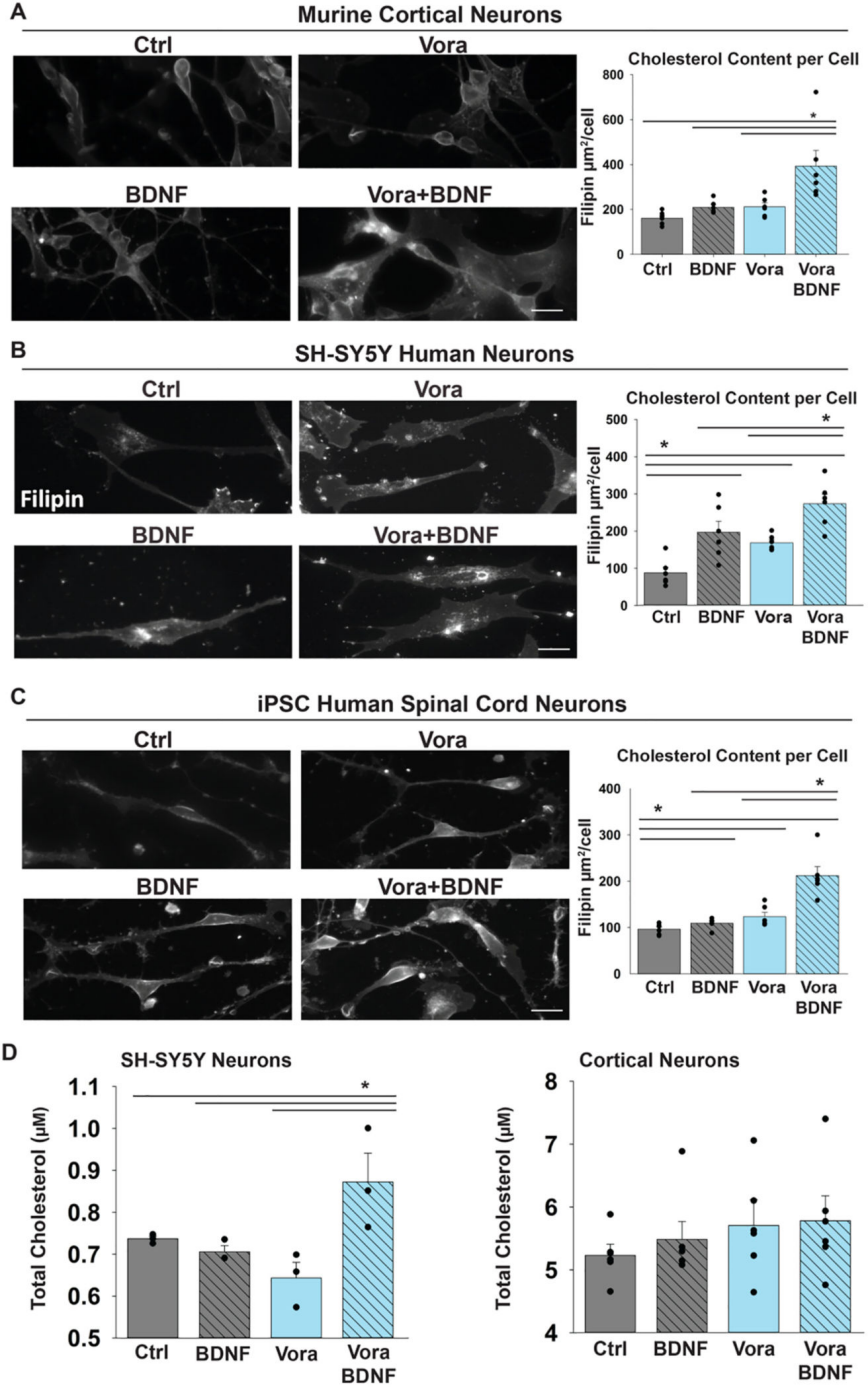
PAR1 inhibition augments BDNF-mediated cholesterol production by murine and human neurons *in vitro*. Filipin staining was used to quantify cholesterol abundance per cell. (A) Human neuronal cell line SH-SY5Y showed increased cholesterol content per cell after 24 h treatment with 1 ng/mL BDNF (*p* = 0.005) or vorapaxar alone (0.02), with combined vorapaxar+BDNF resulting in the greatest increase in cholesterol content (*p* < 0.001), quantified in (B). Similar results were obtained using human iPSC-derived spinal cord neurons treated for 72 h with 50 ng/mL vorapaxar, 1 ng/mL BDNF, or both (C). As in the SH-SY5Y neurons, both vorapaxar (*p* = 0.03) and BDNF (*p* = 0.004) alone increased cellular cholesterol, with the greatest increase observed with combination vorapaxar and BDNF (*p* < 0.001). Combined vorapaxar+BDNF treatment for 72 h also increased neuronal cholesterol content in mouse primary cortical neurons (*p* < 0.01). (D) Total cholesterol content (per well) was also quantified in both SH-SY5Y human neurons and mouse primary cortical neurons after 72 h treatments using the Amplex Red cholesterol assay. In both cell systems, combined vorapaxar+BDNF increased cholesterol, with statistical significance reached in the SH-SY5Y line (*p* = 0.04 compared to control). Statistical significance determined by One-Way ANOVA or One-Way ANOVA on Ranks (cortical neurons) with Student-Newman-Keuls pairwise comparisons, *p < 0.05, *n* = 6 per condition filipin staining, *n* = 3 SH-SY5Y Amplex Red, *n* = 6 cortical neuron Amplex Red. Scale bar = 20 μM.

To confirm that increases in expression of HMGCS1 corresponded to increases in cholesterol production in neuronal cultures, we quantified filipin immunoreactivity as a marker of neuronal cholesterol content (Maxfield and Wustner, 2012) in primary mouse cortical neurons, SH-SY5Y human neurons and human iPSC-derived spinal cord neurons (Fig. 6). Filipin+ area per cell was increased 2-fold by both 24 h treatment with vorapaxar and low-dose BDNF alone (*n* = 6, total *df* = 23, *p* = 0.01 and 0.005, respectively, One-Way ANOVA) and even further increased by the combination of vorapaxar and BDNF to a 3-fold increase relative to control (*p* < 0.001) in SH-SY5Y neurons (Fig. 6B). Similarly, 72 h treatment with either vorapaxar or BDNF alone significantly increased cholesterol staining density (*n* = 6 each, total *df* = 23, with 1.1-fold increase, *p* = 0.004 for BDNF, 1.3-fold increase, *p* = 0.03 for vorapaxar, One-Way ANOVA) in human iPSC neurons. Again, the greatest increase in cholesterol staining was observed with combined vorapaxar-BDNF treatment, more than doubling filipin+ signal compared to vehicle (*p* < 0.001) (Fig. 6C). These results were also replicated in the mouse primary cortical neurons, where again combined vorapaxar+BDNF for 72 h increased cholesterol density 2-fold (Fig. 6A, *n* = 6 each, *df* = 3, *p* < 0.001, One-Way ANOVA on Ranks). As a secondary method of cholesterol quantification, we extracted lipids from both SH-SY5Y neurons and mouse primary neurons. Total cholesterol content was quantified using Amplex Red fluorometric assay. In both cell culture systems, vorapaxar+BDNF increased cholesterol content to 110% of control (Fig. 6D). As neuron culture conditions did not include cholesterol supplementation, any cholesterol present would be derived by *de novo* synthesis. Together, these results highlight that PAR1 inhibition directly increases expression of neuronal machinery necessary for cholesterol production and that this manifests as increased cholesterol production.

### 3.4. Neurite outgrowth and synaptogenesis in vitro mediated by a PAR1 inhibitor-BDNF cocktail is dependent on cholesterol production

**Fig. 7.**
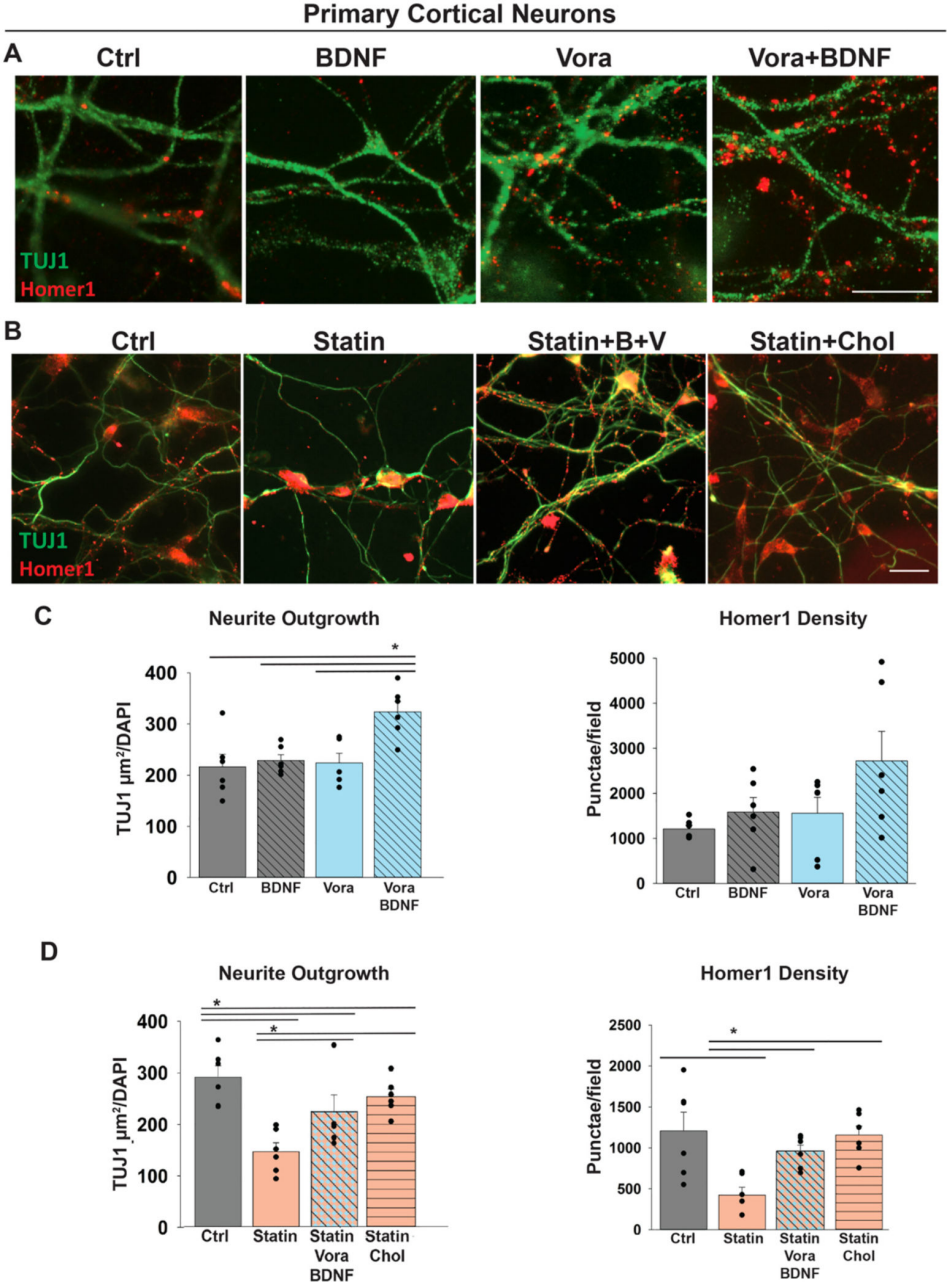
Augmented BDNF-mediated neurite outgrowth after neuronal PAR1 inhibition *in vitro* is dependent on cholesterol production. Immunofluorescent images (A) show staining for TUJ1 and Homer1 in primary murine cortical neurons treated with BDNF (1 ng/mL), Vorapaxar (100 nM) or a combination for 72 h. Histograms (C) show PAR1 inhibition potentiated the effect of subtherapeutic doses of the neurotrophin BDNF, and accelerated neurite production (*p* = 0.004). Similarly, the combination of BDNF and PAR1 inhibition results in increased synapse density at 72 h (N.S.). Immunofluorescent images (B) and associated histogram (D) show reductions in staining for TUJ1 in primary murine cortical neurons treated with a competitive inhibitor of HMGCR, (atorvastatin, 10 nM) for 72 h (*p* = 0.001). Statin-mediated inhibition of neurite outgrowth was partially rescued by supplementation with exogenous cholesterol (10 μM) in the culture media (*p* = 0.002 compared to statin, *p* = 0.03 compared to control), or with the addition of the PAR1 inhibitor Vorapaxar (100 nM) along with 1 ng/mL BDNF (*p* = 0.002 compared to statin, *p* = 0.02 compared to control). Both combined treatment with Vorapaxar and BDNF and replacement of cholesterol also rescue synapse production, measured by Homer1 punctae density (*p* < 0.001 and *p* = 0.01, respectively). Statistical significance determined by One-Way ANOVA (A,C) or One-Way ANOVA on Ranks (B,D) with Student-Newman-Keuls pairwise comparisons, *p < 0.05, *n* = 6 per condition. Scale bar = 10 μM in A, 20 μM in B.

Next we tested the hypothesis that the ability of PAR1 to regulate cholesterol production in primary cortical neurons plays an essential role in their ability to extend neurites (Fig. 7). First, we demonstrated that 10 nM atorvastatin (Lipitor), a commonly used competitive inhibitor of 3-Hydroxy-3-Methylglutaryl-CoA Reductase (HMGCR), significantly limited neurite extension by primary cortical neurons (*n* = 6 each, *df* = 3, *p* = 0.001, ANOVA on ranks) and their production of Homer1 (*n* = 6 each, *df* = 3, *p* < 0.001, ANOVA on ranks). Notably, the roughly 2-fold reduction in neurite outgrowth elicited by atorvastatin at 72 h was partially prevented by addition of exogenous cholesterol (*n* = 6 each, *df* = 3, *p* = 0.002, ANOVA on ranks) to the culture media or by the addition of the PAR1 inhibitor vorapaxar in combination with low-dose BDNF (*n* = 6 each, *df* = 3, *p* = 0.002, ANOVA on ranks) (Fig. 7B,D). Similarly, either combination treatment with vorapaxar and low-dose BDNF, or cholesterol supplementation, was sufficient to restore normal levels of Homer+ staining *in vitro* (*n* = 6 each, *df* = 3, *p* < 0.001 and *p* = 0.01, respectively, One-Way ANOVA on Ranks). In these biosassays, treatment of primary cortical neurons with a low dose of BDNF (1 ng/mL) or vorapaxar alone did not accelerate neurite outgrowth or increase Homer1. Co-application of the PAR1 inhibitor vorapaxar plus BDNF (1 ng/mL) however, did result in a 1.6-fold increase in neurite outgrowth at 72 h, (*n* = 6 each, total *df* = 23, *p* = 0.004, One-Way ANOVA) and a trend to increased Homer1 puncta density (2.3-fold, *n* = 6 each, total *df* = 23, *p* = 0.17), (Fig. 7A,C). Together, these findings suggest that the ability of PAR1-inhibition to enhance BDNF-mediated neurite outgrowth and signs of synapse formation in cell culture is linked to cholesterol production.

### 3.5. PAR1 inhibition augments BDNF-mediated neurite outgrowth in a cholesterol dependent manner

**Fig. 8.**
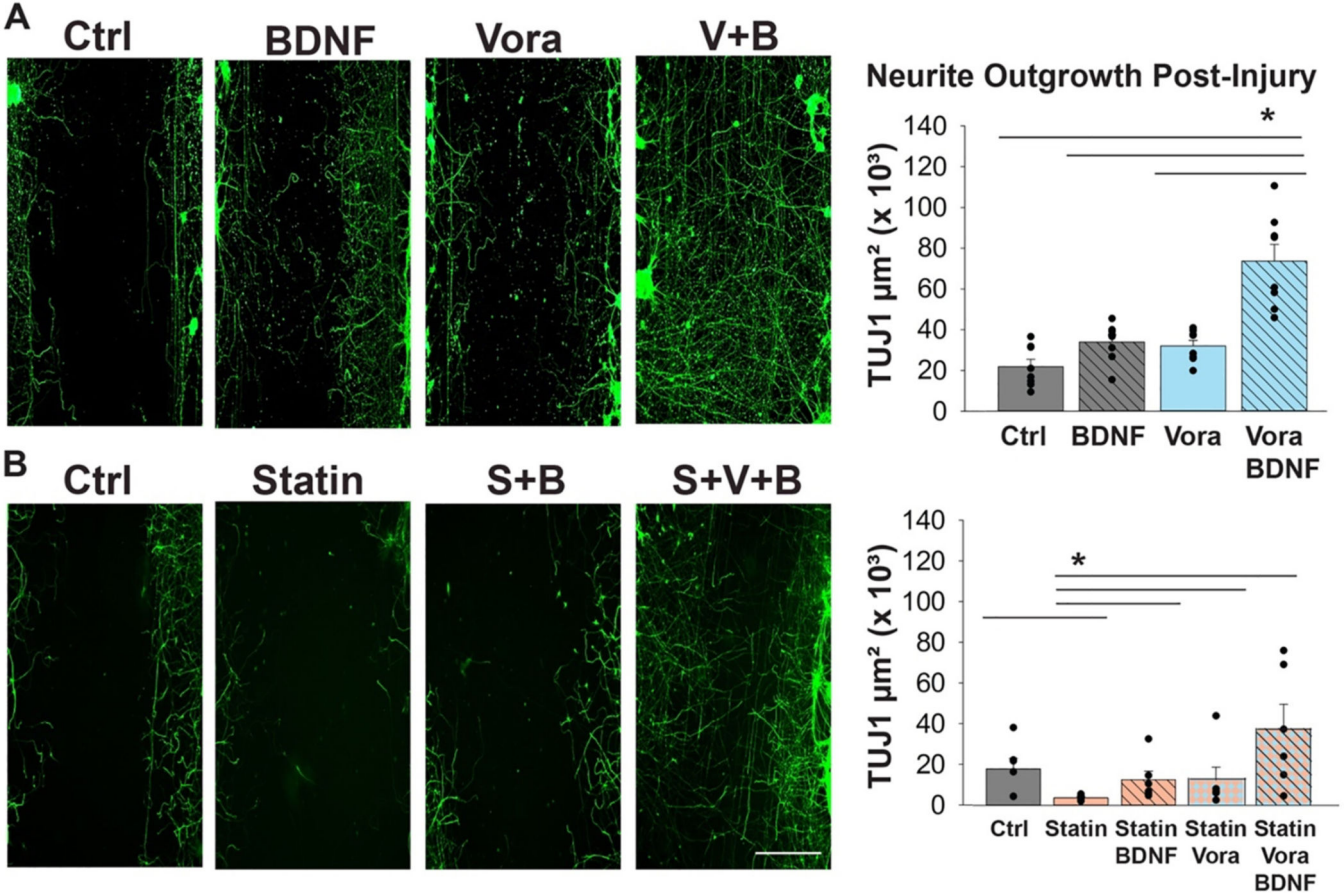
PAR1 knockout enhances BDNF-mediated repair in an *in vitro* model of neural injury in a cholesterol dependent manner. (A) Immunofluorescence images and associated histogram shows murine cortical neurons that were cultured for 5 DIV, then mechanically scratched and allowed to repair for an additional 5 DIV with the addition of the PAR1 inhibitor vorapaxar (Vora, 100 nM), low-dose BDNF (1 ng/mL), or both. Inhibition of PAR1 sensitized neurons to the effects of low-dose BDNF, significantly increasing growth of new neurites through the injury zone, measured by TUJ1+ area in gap (*p* < 0.001 for Vora+BDNF compared to other treatment groups). (B) Images and associated histogram show inhibition of cholesterol production by statin (atorvastatin, 10 nM) diminished neurite outgrowth following mechanical injury (*p* = 0.01 compared to control), and this was reversed by co-treatment with low-dose BDNF (1 ng/mL) or vorapaxar (100 nM) alone (*p* < 0.001). In combination, vorapaxar and BDNF also increased TUJ1 recovery even in the presence of statin (*p* = 0.005) though only reaching approximately 50% of the repair achieved by Vora+BDNF alone. n = 6 for all groups, derived from two independent cell culture preparations. Statistical significance was determined by One-Way ANOVA on Ranks with Student-Newman-Keuls pairwise comparisons, *p < 0.05. Scale bar = 100 μM.

To determine the significance of PAR1 to neurite growth in the context of injury and the potential link to BDNF and cholesterol, we next investigated the impact of PAR1 inhibition in a cortical neuron *in vitro* scratch bioassay (Fig. 8). First, 5 DIV cortical neuron cultures were scratched followed by application of the PAR1 inhibitor vorapaxar (100 nM). After an additional 5 DIV, quantification of TUJ1 immunofluorescence showed that vorapaxar treatment alone only slightly augmented neurite restoration (Fig. 8A, *n* = 8 each, *df* = 3, *p* = 0.53, One-Way ANOVA). We next tested any combinatorial effects of subtherapeutic, low dose of BDNF (1 ng/mL) as we documented in neurite growth under non-injured conditions. While low-dose BDNF alone also had only a slight impact on neurite restoration into the scratch, combined with vorapaxar, low-dose BDNF resulted in a 3-fold increase in TUJ1+ neurites in the injury zone (Fig. 8A, *n* = 8 each, *df* = 3, *p* < 0.001, One-Way ANOVA).

To investigate the potential dependence of the pro-repair effects of vorapaxar and BDNF toward cortical neurons in a cell culture scratch bioassay on cholesterol, we next determined their ability to overcome the effects of statin-mediated inhibition of cholesterol synthesis (Stancu and Sima, 2001) (Fig. 8B). First, treatment of 5 DIV wounded cultures with 10 nM atorvastatin, completely blocked even the residual restoration of TUJ1 neurites observed with vehicle alone (mean = 0.50 relative to control, *n* = 6 each, *df* = 4, *p* = 0.01, One-Way ANOVA). Neither low-dose BDNF nor vorapaxar alone restored neurites in the scratch zone when applied alone, or in the presence of statin (Fig. 8B). However, in combination, treatment of wounded cultures with vorapaxar along with low-dose BDNF drove significant increases in neurite restoration even in the presence of statin (Fig. 8B, *n* = 6 each, *df* = 4, *p* = 0.005, One-Way ANOVA). The addition of statin to the vorapaxar+BDNF treated cultures reduced re-establishment of neurites in the injury zone by approximately 50% (*n* = 6 each, *df* = 10, *t* = 2.49, *p* = 0.03, two-tailed Student’s *t*-test comparing vorapaxar+BDNF with and without statin). These findings suggest that statins can have a significant negative impact on neurite restoration in an *in vitro* injury bioassay. Also, these findings suggest that the ability of the PAR1 inhibitor-BDNF cocktail to promote neurite restoration is dependent on cholesterol production.

### 3.6. Neurite outgrowth inhibitory effects of PAR1 activation are overcome by Gαi inhibition

**Fig. 9.**
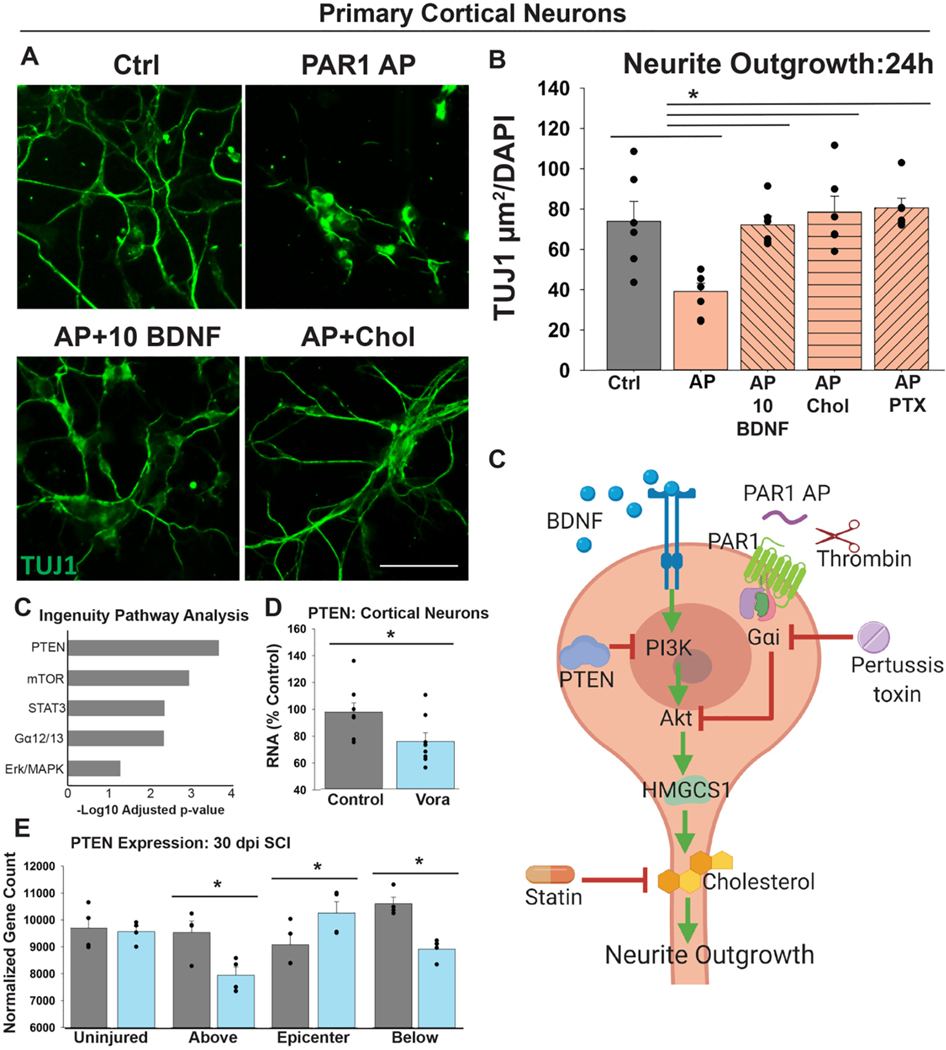
Model for the regulation of neuronal cholesterol production and neurite outgrowth through an interplay between PAR1 and BDNF. (A) Immunofluorescent images and associated histogram (B) show PAR1-activation by PAR1-AP (100 μM) reduces neurite outgrowth (*p* = 0.002) with deleterious effects prevented by addition of 10 ng/mL BDNF alone or 10 μM cholesterol. The negative effect of PAR1-AP on neurite outgrowth was also blocked by addition of 10 nM pertussis toxin, an antagonist of Gαi signaling (*p* = 0.001), which we hypothesize unmasks the ability of BDNF to drive PI3K, Akt and subsequent cholesterol production and neurite outgrowth (*p* < 0.001, each condition compared to PAR1-AP alone, One-Way ANOVA with Student-Newman-Keuls pairwise comparisons, *n* = 6 for all groups, derived from two independent cell culture preparations, Scale bar = 50 μM). (C) Ingenuity pathway analysis of differentially regulated signaling pathways in intact spinal cord reveals that the PTEN signaling pathway is the most differentially expressed between wild type and PAR1 knockouts. (D) Inhibition of PAR1 in primary cortical neuron cultures by application of 100 nM vorapaxar resulted in decreased PTEN expression (*p* = 0.03, Student’s two-tailed t-test, *n* = 4 from two independent cell culture preparations). (E) RNA sequencing of intact and 8 g FEJOTA clip injured spinal cord also demonstrated significant decreases in PTEN expression above and below the site of injury at 30 dpi (*p* = 0.005 and *p* = 0.003, respectively), with increases at the epicenter (*p* = 0.03) (Two-Way Repeated Measures ANOVA, *n* = 4 female mice for each genotype). (F) Proposed mechanism for the influence of PAR1 inhibition on neurite outgrowth. PAR1 activation negatively impacts neurite outgrowth by impeding BDNF signaling. In contrast, when PAR1 is inhibited (by gene knockout or vorapaxar), BDNF is disinhibited and proceeds to activate TrkB increasing neuronal cholesterol synthesis machinery such as HMGCS1, cholesterol synthesis and consequently accelerating neurite outgrowth in the context of development or repair. Findings to date support a model in which blocking PAR1 relieves two potential pathways of inhibition of the BDNF signaling pathway, that is PTEN and Gαi, which in turn can improve both cholesterol production and the potential for neurite growth. Additional studies are needed to validate key aspects of this emerging model.

Since inhibition of PAR1 promoted expression of cholesterol synthesis machinery and neurite outgrowth, we next investigated whether PAR1 over-activation would have the opposite effect. Application of a PAR1-specific activating peptide (PAR1-AP) reduced neurite outgrowth by 1.4-fold at 24 h (Fig. 9A–B, *n* = 6 each, total *df* = 29, *p* = 0.002, One-Way ANOVA). Inhibition of neurite outgrowth by PAR1 activation was overcome by application of 10 ng/mL BDNF, highlighting the opposing actions of BDNF and PAR1 activation. Notably, low-dose 1 ng/mL BDNF was insufficient to overcome the deleterious effects of PAR1-AP (data not shown). Supplementation of the culture media with exogenous cholesterol also overcame the deleterious effects of PAR1-AP (Fig. 9A–D, *n* = 6 each, total *df* = 29, *p* < 0.001, One-Way ANOVA). Together, these complementary findings suggest that the level of PAR1 activation can regulate cholesterol synthesis and neurite outgrowth through a mechanism that includes an interplay with growth factors such as BDNF.

To address the potential signaling mechanisms by which PAR1 activation may inhibit neurite outgrowth, we used pertussis toxin (PTX), which catalyzes ADP-ribosylation of Gαi-bound GPCRs, to inhibit Gi-coupled intracellular signaling, including activation of adenylate cyclase (Snabaitis et al., 2005). Like BDNF, PTX inhibition of Gαi also negated the impact of PAR1 activation, restoring normal neurite outgrowth (*n* = 6 each, total *df* = 29, *p* < 0.001, One-Way ANOVA).

Ingenuity pathway analysis of signaling pathways differentially regulated between PAR1+/+ and PAR1−/− intact spinal cords further indicated that Phosphatase and tensin homolog (PTEN) pathway was the most differentially expressed with loss of PAR1 (Fig. 9C). This was of particular interest given the abundance of evidence indicating downregulation of PTEN improves intrinsic neuronal regenerative capacity and repair after SCI (Christie et al., 2010; Danilov and Steward, 2015; Du et al., 2015; Ohtake et al., 2015; Park et al., 2008). In primary cortical neurons, 72 h treatment with 100 nM vorapaxar decreased PTEN expression by qRT-PCR by 30% (Fig. 9D, *n* = 8 each, *df* = 14, *t* = 2.346, *p* = 0.03, two-tailed Student’s *t*-test). At 30 d after 8 g FEJOTA clip SCI, PTEN expression was also significantly lower both above and below the injury site in PAR1−/− spinal cords (Fig. 9E, *n* = 6–7, total *df* = 23, *p* = 0.005 Above, *p* = 0.03 Below, Two-Way Repeated Measures ANOVA), corresponding with the regions of increased neuronal HMGCS1 expression.

Together, these observations support a model for the potential mechanism(s) by which activity of the PAR1 receptor modulates cholesterol synthesis and neurite growth. We suggest that PAR1 activation by proteases (such as thrombin that are abundant after neural injury) is inhibitory toward the effects of pro-regenerative growth factors (*e.g.* BDNF), in part through a Gαi-dependent mechanism of action (Fig. 9F). In contrast, when PAR1 activity is blocked, this disinhibits BDNF signaling, allowing it to promote PI3K and AKT signaling unopposed, resulting in increased production of cholesterol and acceleration of neurite production. Given the ability of PTEN to inactivate PI3K signaling, the reductions in PTEN we observe with PAR1 inhibition may also play a role the ability of this receptor to regulate neuronal cholesterol production and neurite growth.

## 4. Discussion

PAR1 is densely expressed by CNS neurons and when blocked limits neural injury and fosters functional recovery (Radulovic et al., 2016), yet little is known regarding neuron-specific mechanisms. Through an investigation of the impact of PAR1 loss-of-function on neurite growth *in vitro* and the response of neurons to injury *in vivo* and *in vitro*, our findings highlight a significant role for PAR1 in regulating neurite extension through its ability to control sensitivity to BDNF and dynamically regulate cholesterol biosynthesis.

### 4.1. Blocking PAR1 differentially regulates cholesterol biosynthesis across the astroglial and neuron compartments of the intact and injured spinal cord

Genome-wide sequencing of the spinal cord of wild type and PAR1 knockout mice highlights a novel role for PAR1 as a regulator of CNS lipidogenesis, with cholesterol biosynthesis being the top upregulated Panther Go term. HMGCS1 and HMGCR, which encode rate-limiting enzymes, were among the 17 genes involved in cholesterol biosynthesis upregulated in PAR1 knockouts. Identifying new players in CNS cholesterol production is important since cholesterol is a major constituent of all neural membranes and an essential component of synapse and dendrite formation (Mauch et al., 2001; Pfrieger, 2003). Therefore, we investigated whether increases in cholesterol production in PAR1 knockouts are positioned to contribute to the neurobehavioral improvements we and others recently documented in models of SCI (Radulovic et al., 2016; Whetstone et al., 2017; Yoon et al., 2020).

Within the CNS, cholesterol expression is high in neurons early developmentally, then in oligodendroglia during myelination, and finally in astrocytes in adulthood (Lopes-Cardozo et al., 1986; Saito et al., 1987). Since there is limited ability of cholesterol-rich lipoproteins to cross the blood brain barrier, mature neurons become dependent on neighboring astrocytes for cholesterol delivery (Ferris et al., 2017; Goritz et al., 2002; Mauch et al., 2001; Pfrieger, 2003; Vance et al., 2005). Supporting these observations, we observed astrocytic expression of HMGCS1 in the intact spinal cord and notably, this was two-fold greater in PAR1 knockouts. Consistent with studies in other brain regions, we also observe low levels of HMGCS1 expression by neurons in the intact cord and note again these levels were approximately two-fold higher in PAR1 knockouts. Together, these findings suggest that in the intact CNS that PAR1 is a negative regulator of cholesterol biosynthesis across astrocytes and neurons with PAR1 inhibition increasing HMGCS1 expression in both compartments.

In response to SCI, prominent increases in neuronal HMGCS1 expression occurred in spinal segments above and below the injury epicenter across genotypes, highlighting the potential importance of neuronal cholesterol to the injury and repair response. Supporting the negative regulatory role for PAR1 in neuronal cholesterol production, PAR1 knockout mice showed even greater increases in neuronal HMGCS1 after SCI. Whole genome RNA-sequencing confirmed HMGCS1 increases in PAR1 knockouts and highlighted additional increases in many other cholesterol biosynthesis enzymes. That these findings translate to human disease is suggested by dense HMGCS1 expression by spinal cord neurons before and after SCI.

While HMGCS1 expression in neurons was increased after SCI, expression by spinal cord astrocytes was reduced. PAR1−/− astrocytes trended to increased HMGCS1 expression relative to wild type following injury, but levels remained reduced relative to intact cord. The differential regulation of HMGCS1 across the neuron and astrocyte compartments of the intact and injured cord may reflect the multiple roles astrocytes take post-injury. Similar reductions in astrocyte cholesterol occur in other neural injury models (Itoh et al., 2018; Orre et al., 2014; Valenza et al., 2010), with strategies to prevent this providing functional benefit (Tassoni et al., 2019). Altogether, these findings suggest that neurotrauma re-engages neuronal HMGCS1 expression, perhaps reverting to an earlier developmental state, with increases augmented by blocking PAR1. Hence, PAR1 inhibition should be investigated as a strategy to reduce neuronal dependence on astrocyte cholesterol during critical periods of neural injury and repair.

### 4.2. Link between improved neurobehavioral recovery in PAR1 knockout mice after SCI and increases in neuronal cholesterol biosynthesis machinery

Our continued interest in dissecting mechanisms of PAR1 action in the intact and injured CNS is driven by our discovery that mice with constitutive PAR1 gene knockout show improved recovery after SCI. For example, compared to wild types, PAR1 knockouts show improved sensorimotor function, including a two-fold increase in BMS scores and stepping accuracy (see (Radulovic et al., 2016)). These findings in the FEJOTA clip model of severe contusion-compression injury were verified in a thoracic impact model (Whetstone et al., 2017) and here after lateral compression SCI. We provide new evidence that enhanced sensorimotor recovery after SCI in PAR1 knockouts is accompanied by improvements in the appearance of neurofilament+ axons in the injury epicenter and below. Also, the number of neurons expressing HMGCS1 above the injury epicenter correlated positively with recovery of sensorimotor function and with neurofilament area below the epicenter. These findings are exciting because they demonstrate that blocking PAR1 improves neurological function and the substrates for repair. Whether blocking PAR1 selectively in neurons after SCI is sufficient to enhance recovery will be an important line of future research. These data do not suggest that cholesterol is solely responsible for the improved motor outcomes we observe in subjects lacking PAR1, and in these model systems we are unable to distinguish between axonal regeneration, local sprouting, circuit reorganization, or other processes which are known to contribute to the overall repair process. Additional research will be necessary to further understand the impact of PAR-signaling and lipid availability on these distinct avenues, as well as related factors, such as inflammation or myelin repair.

### 4.3. Neurotrophic coupling between PAR1 and BDNF accelerates neurite outgrowth in a cholesterol dependent manner

BDNF is a powerful neurotrophic factor that promotes neurite outgrowth developmentally and nerve sprouting/regeneration in adulthood (Mamounas et al., 2000; Rabacchi et al., 1999). BDNF also supports neuron survival across the lifespan (Baydyuk and Xu, 2014; Kelly-Spratt et al., 2002; Middlemas et al., 1999). The limited availability of growth factors such as BDNF in the mature CNS, and further reductions after injury, are contributing factors to limited repair (Conner et al., 1997; Garraway and Huie, 2016). Here we document that blocking PAR1 enhances the neurotrophic effects of suboptimal BDNF. That this new neurobiological principle can be harnessed to improve nerve regeneration is supported by observations that the PAR1 inhibitor-BDNF cocktail not only improved neurite outgrowth, but also increased establishment of new neurites in a cortical neuron scratch bioassay. Taken together, these findings suggest PAR1 activation serves as a brake on the ability of BDNF to promote neurite growth and regeneration.

Highlighting the potential translatability of the PAR1 inhibitor-BDNF cocktail to human disease, the pro-neurite growth effects toward murine cortical neurons were mirrored in SH-SY5Y human neuron bioassays. While a role for PAR1 in limiting growth factor signaling in the adult CNS could play a role in homeostasis, after injury, when PAR1-activating proteases such as thrombin, plasmin and kallikrein 6 are elevated (Radulovic et al., 2013; Radulovic et al., 2016; Yoon et al., 2013), the PAR1-BDNF growth inhibitory interaction likely impedes repair. The fundamental role for PAR1-inhibition in improving sensitivity of neurons to BDNF is also supported by our recent demonstration of parallel neurobiology in myelinating glia, where inhibition of oligodendrocyte progenitor PAR1 enhances BDNF-mediated expression of myelin proteins (Yoon et al., 2020). Additional efforts are needed to determine if blocking PAR1 also improves responses to other growth factors with such findings pointing to an important strategy to enhance repair.

Our discovery that PAR1 is a negative regulator of the neuronal cholesterol biosynthesis machinery, while knowing that BDNF is a positive regulator (Suzuki et al., 2007), led us to hypothesize that cholesterol biosynthesis is an essential part of the PAR1-BDNF trophic coupling mechanism. To test this, we first demonstrated that reductions in neurite outgrowth triggered by PAR1-activation (Yoon et al., 2013) were at least partially prevented by BDNF. Next we documented that inhibition of neurite outgrowth by PAR1-activation was also overcome by adding exogenous cholesterol. Moreover, the PAR1 inhibitor-BDNF combination enhanced cholesterol production in human SH-SY5Y and iPSC-derived neurons. Finally, the PAR1 inhibitor-BDNF cocktail attenuated the deleterious effects of atorvastatin, an inhibitor of cholesterol biosynthesis, on neurite replacement in a scratch bioassay. Each of these lines of evidence suggests that PAR1 and BDNF reciprocally regulate neuronal cholesterol production and that blocking PAR1 enhances neurite outgrowth in a cholesterol-dependent manner.

A full understanding of the intracellular signaling mechanisms mediating the neurite inhibitory effects of PAR1 will require additional efforts, but we provide evidence of a functional link to Gαi. For example, the addition of pertussis toxin to primary cortical neuron cultures disables the neurite inhibitory action of PAR1 activation. Since many growth factors, including BDNF, signal their growth promoting effects in part by activation of adenylate cyclase, it is possible that engagement of Gαi by PAR1-activation suppresses growth factor-mediated neurite growth.

It is also noteworthy that inhibition of PAR1 downregulates expression of PTEN and leads to differential expression of the PTEN signaling pathway in the injured spinal cord. PTEN is a master regulator of intrinsic neuronal regenerative capacity, and inhibition of PTEN promotes axon regeneration (Abiraman et al., 2015; Christie et al., 2010; Danilov and Steward, 2015; Ohtake et al., 2015; Park et al., 2008). It is therefore possible that the reductions we observe in PTEN with PAR1 inhibition *in vitro* and *in vivo*, partially account for improved neurobehavioral measures of spinal cord repair. The mechanism through which PAR1 signaling intersects with PTEN activation is clear, and this important observation with implications for neural repair strategies warrants additional follow-up.

## 5. Conclusions

In sum, mice with constitutive PAR1 knockout show increases in neuronal HMGCS1, axonal and synaptic proteins after SCI. Coupled with this, selective blockade of neuronal PAR1 increased neurite growth and improved neurite replacement in an *in vitro* neural injury assay, each in a cholesterol-dependent manner. These signs of r repair were accompanied by reductions in PTEN, a well-studied inhibitor of nerve regeneration *in vivo* and *in vitro*. Moreover, PAR1 inhibition sensitized neurons to the pro-regenerative effects of BDNF, increasing neurite outgrowth *in vitro* in a cholesterol-dependent manner. The significance of these newly identified roles for PAR1 inhibition in improving the response of the CNS to injury is underscored by the existence of FDA-approved PAR1 small molecule inhibitors that could be repurposed to enhance cholesterol production, growth factor sensitivity and CNS regenerative repair mechanisms.

## Supplementary Material

mmc1

## Figures and Tables

**Table 1:** Primary antibodies used across *in vivo* and *in vitro* studies.

Target	Dilution	Co, Cat. #	RRID
GFAP	1:2000	Abcam, ab4674	AB_304558
HMGCS1	1:1000	Thermo Fischer, PA5–29488	AB_2546964
NeuN	1:1000	Milllipore, MAB377	AB_2298772
NeuN	1:1000	Abcam, ab177487	AB_2532109
Neurofilament	1:1000	Abcam, ab4680	AB_304560
PAR1	1:100	Santa Cruz, sc-13,503	AB_2101175
TUJ1	1:1000 (mouse) 1:500 (human)	Millipore, AB9354	AB_570918

## Footnotes

1 Studies involving RNAsequencig and immunostaining were performed as an extension of neurobehavioral studies regarding the impact of PAR1−/− on motor function following spinal cord injury. Therefore, data representing the outcomes from ladder walk and incline plane testing (Fig. 4 C–D) are reproduced here from our earlier publication (Radulovic, et al., Neurobiol Dis. 2016 September 93: 226–242).

## Abbreviations:

BBB: blood-brain barrier
BDNF: brain derived neurotrophic factor
BMS: Basso Mouse Scale
GFAP: glial fibrillary acidic protein
HDL: high density lipoprotein
HMG CoA: β-Hydroxy β-methylglutaryl-CoA
HMGCR: HMG CoA Reductase
HMGCS1: HMG CoA Synthase
iPSC: induced pluripotent stem cell
LC: lateral compression
NS: not significant
PTEN: phosphatase and tensin homolog
SCI: spinal cord injury
