# Supplementary material for: The thrombin receptor links brain derived neurotrophic factor to neuron cholesterol production, resiliency and repair after spinal cord injury
Source: Neurobiol Dis. Author manuscript; Available in PMC 2021 May 1. (PMC8021459; doi:10.1016/j.nbd.2021.105294)
Supplement: mmc1 [file NIHMS1670620-supplement-mmc1.docx]

| **ABOVE** |  |  | **Normalized Gene Count:**  **Mean (±SEM)** | |
| --- | --- | --- | --- | --- |
| **Gene** | **logFC** | **FDR** | **PAR1+/+** | **PAR1-/-** |
| Hmgcs1 | -0.243 | **0.011** | 23709 (925.5) | 20041 (505.8) |
| Sqle | -0.090 | 0.345 | 6387 (121.9) | 5999 (98.3) |
| Mvd | 0.096 | 0.503 | 1505 (59.0) | 1611 (74.4) |
| Lss | -0.401 | **0.0001** | 5313 (204.0) | 4025 (198.1) |
| Msmo1 | 0.101 | 0.324 | 6438 (238.4) | 6904 (128.2) |
| Dhcr7 | 0.136 | 0.191 | 3061 (49.5) | 3362 (110.2) |
| Idi1 | 0.015 | 0.907 | 4584 (207.5) | 4632 (72.6) |
| Dhcr24 | -0.003 | 0.983 | 14339 (293.3) | 14310 (399.4) |
| Ebp | 0.276 | **0.040** | 1434 (80.0) | 1735 (79.4) |
| Mvk | 0.215 | 0.073 | 1886 (24.6) | 2188 (122.0) |
| Fdps | 0.180 | 0.076 | 3243 (72.1) | 3672 (120.8) |
| Sc5d | -0.230 | **0.030** | 5190 (233.9) | 4428 (157.1) |
| Hmgcr | -0.492 | **2.2*10^-6^** | 5671 (262.9) | 4032 (170.6) |
| Pmvk | 0.204 | 0.066 | 2510 (93.9) | 2891 (104.0) |
| Fdft1 | 0.173 | 0.068 | 7437 (209.7) | 8385 (238.1) |
| Nsdhl | 0.120 | 0.304 | 2213 (78.9) | 2405 (67.8) |
| Hsd17b7 | -0.352 | **0.0005** | 2891 (107.9) | 2265 (24.2) |
|  |  |  |  |  |
| **EPICENTER** | |  | **Normalized Gene Count:**  **Mean (±SEM)** | |
| **Gene** | **logFC** | **FDR** | **PAR1+/+** | **PAR1-/-** |
| Hmgcs1 | 0.278 | 0.951 | 11325 (308.7) | 13732 (778.6) |
| Sqle | 0.180 | 1.000 | 2867 (79.2) | 3249 (378.1) |
| Mvd | -0.187 | 1.000 | 1053 (63.6) | 925 (125.4) |
| Lss | 0.029 | 1.000 | 2943 (108.1) | 3003 (143.4) |
| Msmo1 | 0.092 | 1.000 | 3564 (136.0) | 3799 (428.1) |
| Dhcr7 | 0.086 | 1.000 | 1980 (113.6) | 2102 (147.1) |
| Idi1 | 0.246 | 1.000 | 2125 (73.5) | 2520 (278.5) |
| Dhcr24 | 0.203 | 1.000 | 5913 (602.2) | 6809 (1074.9) |
| Ebp | 0.089 | 1.000 | 1503 (66.9) | 1598 (11.7) |
| Mvk | -0.211 | 1.000 | 1454 (105.7) | 1257 (191.5) |
| Fdps | 0.154 | 1.000 | 1802 (151.3) | 2005 (350.6) |
| Sc5d | 0.184 | 1.000 | 2666 (58.4) | 3029 (154.0) |
| Hmgcr | 0.149 | 1.000 | 3340 (160.6) | 3702 (155.7) |
| Pmvk | -0.178 | 1.000 | 2089 (132.0) | 1847 (166.3) |
| Fdft1 | -0.084 | 1.000 | 6344 (208.7) | 5987 (363.3) |
| Nsdhl | -0.015 | 1.000 | 1478 (58.9) | 1463 (200.0) |
| Hsd17b7 | -0.005 | 1.000 | 1845 (139.0) | 1839 (77.1) |
|  |  |  |  |  |
| **BELOW** |  |  | **Normalized Gene Count:**  **Mean (±SEM)** | |
| **Gene** | **logFC** | **FDR** | **PAR1+/+** | **PAR1-/-** |
| Hmgcs1 | 0.086 | 0.830 | 12733 (1389.5) | 13515 (1240.9) |
| Sqle | 0.230 | 0.444 | 3251 (296.4) | 3813 (296.2) |
| Mvd | 0.303 | 0.369 | 802 (71.1) | 990 (64.4) |
| Lss | -0.138 | 0.665 | 3471 (356.3) | 3154 (156.2) |
| Msmo1 | 0.294 | 0.307 | 3452 (298.7) | 4231 (346.7) |
| Dhcr7 | 0.128 | 0.679 | 2016 (118.3) | 2203 (80.9) |
| Idi1 | 0.392 | 0.226 | 2243 (274.3) | 2945 (320.0) |
| Dhcr24 | 0.377 | 0.395 | 7285 (1637.3) | 9461 (951.7) |
| Ebp | 0.254 | 0.387 | 1283 (67.6) | 1530 (59.3) |
| Mvk | 0.487 | 0.113 | 1094 (112.3) | 1534 (103.7) |
| Fdps | 0.468 | 0.151 | 1666 (225.1) | 2305 (208.7) |
| Sc5d | -0.036 | 0.922 | 3365 (216.5) | 3283 (210.6) |
| Hmgcr | -0.336 | 0.199 | 4257 (305.9) | 3372 (177.1) |
| Pmvk | 0.408 | 0.112 | 1929 (118.4) | 2560 (74.1) |
| Fdft1 | 0.113 | 0.711 | 5874 (260.5) | 6353 (322.6) |
| Nsdhl | 0.297 | 0.343 | 1254 (94.7) | 1541 (116.7) |
| Hsd17b7 | -0.311 | 0.286 | 2083 (154.7) | 1679 (86.5) |

**Supplementary Figure 1 RNAsequencing of injured spinal cord at 30 days post injury.** Tables show raw data for RNAsequencing of spinal cord regions at 30 d following FEJOTA clip (see heatmaps, Fig. 1). Columns show log fold-changes (logFC, positive values show increased expression in PAR1-/- relative to PAR1+/+), FDR-adjusted p-values (p < 0.05 bolded), as well as the mean normalized gene counts (±SEM) for both PAR1+/+ and PAR1-/- subjects. **p < 0.05**, n = 4 for each genotype.
